# Safety and immunogenicity of two heterologous HIV vaccine regimens in healthy, HIV-uninfected adults (TRAVERSE): a randomised, parallel-group, placebo-controlled, double-blind, phase 1/2a study

**DOI:** 10.1016/S2352-3018(20)30229-0

**Published:** 2020-09-30

**Authors:** Lindsey R Baden, Daniel J Stieh, Michal Sarnecki, Stephen R Walsh, Georgia D Tomaras, James G Kublin, M Juliana McElrath, Galit Alter, Guido Ferrari, David Montefiori, Philipp Mann, Steven Nijs, Katleen Callewaert, Paul Goepfert, Srilatha Edupuganti, Etienne Karita, Johannes P Langedijk, Frank Wegmann, Lawrence Corey, Maria G Pau, Dan H Barouch, Hanneke Schuitemaker, Frank Tomaka, Traverse/HVTN 117/HPX2004 Study Team, Julie A. Ake, Susan Buchbinder, Karen Buleza, Kristen W. Cohen, Trevor A. Crowell, Zelda Euler, Ian Frank, Dimitri Goedhart, Michael Keefer, Colleen Kelly, Ken Mayer, Joseph Nkolola, Lauren Peter, Merlin L. Robb, Nadine Rouphael, Lorenz Scheppler, Magda Sobieszczyk, Hong Van Tieu

**Affiliations:** a Brigham and Women's Hospital, Harvard Medical School, Boston, MA, USA; b Janssen Vaccines & Prevention, Leiden, Netherlands; c Janssen Vaccines, Bern, Switzerland; d Beth Israel Deaconess Medical Center, Harvard Medical School, Boston, MA, USA; e Department of Surgery and Duke Human Vaccine Institute, Duke University Medical Center, Durham, NC, USA; f Vaccine and Infectious Disease Division, Fred Hutchinson Cancer Research Center, Seattle, WA, USA; g Ragon Institute of MGH, MIT, and Harvard, Cambridge, MA, USA; h Janssen Infectious Diseases, Beerse, Belgium; i Division of Infectious Disease, Department of Medicine, University of Alabama at Birmingham, Birmingham, AL, USA; j Hope Clinic of the Emory Vaccine Center, Division of Infectious Diseases, Department of Medicine, Emory University School of Medicine, Atlanta, GA, USA; k Rwanda Zambia HIV Research Group, Kigali, Rwanda; l Janssen Research and Development, Titusville, NJ, USA

## Abstract

**Background:**

Bioinformatically designed mosaic antigens increase the breadth of HIV vaccine-elicited immunity. This study compared the safety, tolerability, and immunogenicity of a newly developed, tetravalent Ad26 vaccine with the previously tested trivalent formulation.

**Methods:**

This randomised, parallel-group, placebo-controlled, double-blind, phase 1/2a study (TRAVERSE) was done at 11 centres in the USA and one centre in Rwanda. Eligible participants were adults aged 18 to 50 years, who were HIV-uninfected, healthy at screening based on their medical history and a physical examination including laboratory assessment and vital sign measurements, and at low risk of HIV infection in the opinion of study staff, who applied a uniform definition of low-risk guidelines that was aligned across sites. Enrolled participants were randomly assigned at a 2:1 ratio to tetravalent and trivalent groups. Participants in tetravalent and trivalent groups were then further randomly assigned at a 5:1 ratio to adenovirus 26 (Ad26)-vectored vaccine and placebo subgroups. Randomisation was stratified by region (USA and Rwanda) and based on a computer-generated schedule using randomly permuted blocks prepared under the sponsor's supervision. We masked participants and investigators to treatment allocation throughout the study. On day 0, participants received a first injection of tetravalent vaccine (Ad26.Mos4.HIV or placebo) or trivalent vaccine (Ad26.Mos.HIV or placebo), and those injections were repeated 12 weeks later. At week 24, vaccine groups received a third dose of tetravalent or trivalent together with clade C gp140, and this was repeated at week 48, with placebos again administered to the placebo group. All study vaccines and placebo were administered by intramuscular injection in the deltoid muscle. We assessed adverse events in all participants who received at least one study injection (full analysis set) and Env-specific binding antibodies in all participants who received at least the first three vaccinations according to the protocol-specified vaccination schedule, had at least one measured post-dose blood sample collected, and were not diagnosed with HIV during the study (per-protocol set). This study is registered with Clinicaltrials.gov, NCT02788045.

**Findings:**

Of 201 participants who were enrolled and randomly assigned, 198 received the first vaccination: 110 were in the tetravalent group, 55 in the trivalent group, and 33 in the placebo group. Overall, 185 (93%) completed two scheduled vaccinations per protocol, 180 (91%) completed three, and 164 (83%) completed four. Solicited, self-limiting local, systemic reactogenicity and unsolicited adverse events were similar in vaccine groups and higher than in placebo groups. All participants in the per-protocol set developed clade C Env binding antibodies after the second vaccination, with higher total IgG titres after the tetravalent vaccine than after the trivalent vaccine (10 413 EU/mL, 95% CI 7284–14 886 in the tetravalent group compared with 5494 EU/mL, 3759–8029 in the trivalent group). Titres further increased after the third and fourth vaccinations, persisting at least through week 72. Other immune responses were also higher with the tetravalent vaccine, including the magnitude and breadth of binding antibodies against a cross-clade panel of Env antigens, and the magnitude of IFNγ ELISPOT responses (median 521 SFU/10^6^ peripheral blood mononuclear cells [PBMCs] in the tetravalent group and median 282 SFU/10^6^ PBMCs in the trivalent group after the fourth vaccination) and Env-specific CD4+ T-cell response rates after the third and fourth vaccinations. No interference by pre-existing Ad26 immunity was identified.

**Interpretation:**

The tetravalent vaccine regimen was generally safe, well-tolerated, and found to elicit higher immune responses than the trivalent regimen. Regimens that use this tetravalent vaccine component are being advanced into field trials to assess efficacy against HIV-1 infection.

**Funding:**

National Institutes of Health, Henry M Jackson Foundation for Advancement of Military Medicine and the US Department of Defense, Ragon Institute of MGH, MIT, & Harvard, Bill & Melinda Gates Foundation, and Janssen Vaccines & Prevention.

## Introduction

Current estimates of 37·9 million people living with HIV worldwide and 1·7 million new infections annually,^1^ with no cure on the horizon, make development of an effective prophylactic vaccine a global priority.^2^ Several vaccine development strategies in HIV have been assessed^3^, ^4^, ^5^, ^6^, ^7^ with one showing modest (∼30%) efficacy,^8^, ^9^ although a subsequent study was halted with no evidence of efficacy.^10^ A major challenge in HIV vaccine development is the substantial global genetic diversity of HIV-1,^11^ which limits coverage of both humoral and cellular immune responses to the strains of HIV-1 selected in vaccine regimens.

One strategy to potentially overcome this limited immune coverage is the mosaic HIV-1 vaccine concept,^12^ which uses in-silico design of complementary *env, gag*, and *pol* sequences that are computationally predicted to broaden the immune response and thereby increase the immune coverage against globally circulating HIV-1 strains. Bivalent and trivalent sets of mosaic inserts have been shown to broaden the immune response in non-human primate models.^13^, ^14^, ^15^ We applied this mosaic concept to develop a vaccine to achieve more optimal coverage of circulating HIV-1 strains. A bivalent mosaic strategy consisting of two HIV-1 Gag, Pol, and Env antigens was selected, to balance the competing issues of theoretically broader coverage with multivalent formulations and the practical considerations of manufacturing complexities and potential antigen interactions.^13^

In humans, bivalent mosaic inserts have elicited cross-clade immune responses.^16^ Previous clinical studies have shown that the adenovirus serotype 26 (Ad26) vector is well tolerated and elicits humoral and cellular immune responses,^17^, ^18^, ^19^, ^20^, ^21^ mucosal responses,^22^ and immune responses that are augmented by sequential vaccinations including Env proteins.^17^, ^23^ Mosaic 2 Env has highest coverage of clade C strains of HIV-1, and is designed to complement the coverage of Mosaic 1 Env, which has highest coverage of clade B and CRF01_AE strains. The original Mosaic 2 Env construct showed non-optimal cell surface expression and immune response in preclinical models. Along with some manufacturing challenges, this led to APPROACH,^17^ an initial phase 1/2a study of this Ad26 mosaic gp140 regimen with a trivalent vector combination (Ad26.Mos.HIV) encoding a single Mosaic Env antigen and two Mosaic Gag-Pol antigens (2:1:1 virus particle ratio), rather than a tetravalent vector combination encoding two complementary mosaic sequences per antigen (two Mosaic Env antigens and two Mosaic Gag-Pol antigens; 1:1:1:1 virus particle ratio).

We report a first-in-human, multinational, randomised, placebo-controlled, phase 1/2a clinical study to compare safety and immune responses elicited by a newly developed, tetravalent Ad26 vaccine with the previously tested trivalent formulation.^17^, ^18^

**Research in context**

**Evidence before this study**

There is a need for an effective prophylactic vaccine against HIV-1 infection. A major hurdle in HIV-1 vaccine development is the global diversity of the virus. One strategy to overcome this diversity is the so-called mosaic concept: the use of bioinformatics to design molecular inserts for different target antigens, to broaden the immune response and improve coverage against multiple strains of HIV-1. We used the terms “HIV-1”, “vaccine”, and “mosaic” to do an unrestricted PubMed search on March 31, 2020, which revealed several preclinical and phase 1 clinical studies of vaccination regimens using different viral vectors, including modified vaccinia Ankara and recombinant adenovirus serotype 26 (Ad26). The trivalent Ad26 mosaic vaccine candidate has been shown to be effective in raising immune responses against antigen components in preclinical studies and phase 1/2a clinical studies in human participants. Further work to enhance the breadth of the response has led to a tetravalent vaccine candidate, and to our knowledge our study is the first to assess that candidate in humans.

**Added value of this study**

This is the first-in-human comparison of the trivalent and tetravalent mosaic vaccine candidates that shows that the tetravalent formulation enhances both the magnitude and breadth of humoral and cellular immune responses compared with the trivalent vaccine. These immune responses persisted until the end of follow-up at 72 weeks. Both regimens were generally well tolerated with predominantly mild to moderate, transient reactogenicity and did not raise any safety concerns.

**Implications of all the available evidence**

In preclinical studies in non-human primates, the mosaic vaccine concept has been shown to be protective against heterologous simian HIV challenge. In those models the vaccine induced immune responses that resemble those observed in humans in clinical testing. Although no direct correlation has been shown between such protection and any specific component of the induced immune response, the enhancement of all aspects of those responses in humans by the tetravalent formulation is sufficient justification to take this regimen into further clinical testing in targeted populations. Such studies are now ongoing in young women in South Africa, and in men who have sex with men and transgender individuals in the Americas and Europe.

## Methods

### Study design and participants

This randomised, parallel-group, placebo-controlled, double-blind, phase 1/2a study (TRAVERSE) was done at 11 centres in the USA and one centre in Rwanda from July 12, 2016, until Aug 27, 2018. The protocol was approved by the institutional review boards of each study centre. The study adhered to current Declaration of Helsinki and ICH Good Clinical Practice guidelines. The objective was to compare the safety, tolerability, and immunogenicity of two vaccination regimens consisting of two immunisations with Ad26.Mos.HIV or Ad26.Mos4.HIV at day 0 and week 12, followed by two concomitant immunisations of clade C gp140 and Ad26.Mos.HIV or Ad26.Mos4.HIV at weeks 24 and 48, respectively.

Eligible participants were adults aged 18 to 50 years, who were HIV-uninfected, healthy at screening based on their medical history and a physical examination including laboratory assessment and vital sign measurements, and at low risk of HIV infection in the opinion of study staff, who applied a uniform definition of low-risk guidelines that was aligned across sites (appendix p 2). Full inclusion and exclusion criteria are listed in the appendix (p 2). All participants provided written informed consent.

### Randomisation and masking

Enrolled participants were randomly assigned at a 2:1 ratio to tetravalent and trivalent groups. Participants in tetravalent and trivalent groups were then further randomly assigned at a 5:1 ratio to vaccine and placebo subgroups. Randomisation was stratified by region (USA and Rwanda) and based on a computer-generated schedule using randomly permuted blocks prepared under the sponsor's supervision. We masked participants and investigators to treatment allocation throughout the study. Vaccines and placebo were provided in identical syringes, masked with blinding tape.

### Procedures

On day 0, participants received a first injection of tetravalent vaccine (Ad26.Mos4.HIV), trivalent vaccine (Ad26.Mos.HIV), or placebo, and those injections were repeated 12 weeks later. At week 24, vaccine groups received a third dose of tetravalent or trivalent together with clade C gp140, and this was repeated at week 48, with placebos again administered to the placebo group. All study vaccines and placebo were administered by intramuscular injection in the deltoid muscle; Ad26.Mos4.HIV or Ad26.Mos.HIV and clade C gp140 being given in opposing arms. A schematic of the vaccination schedule and study vaccines is shown in the appendix (p 13).

Soluble HIV envelope antigen based on Mosaic 2 Env has been described previously,^13^ but showed non-optimal cell surface expression and immune response. The Mosaic 2 Env sequence was optimised to have higher expression of correctly folded native-like Env. The modification remained as close as possible to the optimal clade C sequence coverage and, when paired with Mosaic 1 Env (which has highest coverage of clade B and CRF01_AE), conferred optimal global HIV-1 sequence coverage. An optimised soluble Mosaic 2S Env was based on the gp140 ectodomain with or without a fusion with a C-terminal GCN4 trimerisation domain. Similar membrane-anchored versions of the designs were made in which the ectodomains were cleavable by furin, the C-termini were extended with the transmembrane domain, and a short cytoplasmic region truncated after residue 712. Expression of the Mosaic 2S Envs variant was much higher than the Mosaic 2 Env (appendix pp 13–14).

Immunogenicity of candidate alternative vectors was assessed in rabbits, for selection of the optimal candidate to be taken into this clinical evaluation in a vaccine regimen selected from earlier studies.^16^ Preclinical data in rabbits given two doses (5 × 10^9^ or 5 × 10^10^ virus particles) of Ad26.Mos4.HIV increased the magnitude of clade C HIV-specific antibody titres in the absence of negative effects on clade B, indicating that improved gp120-binding antibodies were induced (appendix pp 13–14).

The trivalent Ad26.Mos.HIV vaccine formulation contains 5 × 10^10^ virus particles per 0·5 mL injection consisting of the following in 1:1:2 ratio: Ad26.Mos1.Gag-Pol (replication-incompetent, Ad26-encoding Mos1 and HIV-1 Gag and Pol protein), Ad26.Mos2.Gag-Pol (replication-incompetent, Ad26-encoding Mos2 HIV-1 Gag and Pol protein), and Ad26.Mos1.Env (Mos1 HIV-1 Env protein). The Ad26.Mos4.HIV tetravalent vaccine formulation also contains 5 × 10^10^ virus particles per 0·5 mL injection with the three trivalent components and additionally Ad26.Mos2S.Env in a 1:1:1:1 ratio. The new Ad26.Mos2S.Env component encodes modified Mos2 HIV-1 Env protein. The gp140 component is HIV-1 clade C gp140 strain 97ZA012, which contains recombinant trimeric glycoprotein gp140. All Ad26.Mos components and clade C gp140 are manufactured in PER.C6 cells. Each 0·5 mL dose contained 250 μg total gp140 with 0·425 mg aluminium phosphate adjuvant. Placebo doses were 0·5 mL of 0·9% saline for injection.

### Outcomes

Participants were monitored for 30 min post-injection for immediate reactions. Participants then used diary cards to record solicited local (pain or tenderness, erythema, and swelling or induration) and systemic (pyrexia or fever, fatigue, headache, nausea, myalgia, and chills) adverse events on the day of injection and for the subsequent 7 days. Further safety assessments included clinical laboratory tests (haematology, chemistry, urinalysis), vital sign measurements, and physical examinations. Unsolicited adverse events were recorded until 28 days after each study injection. Serious adverse events, adverse events leading to discontinuation from the study, and adverse events of special interest such as confirmed HIV infection, were recorded through week 72. Adverse events and clinical laboratory values were graded according to the National Institute of Allergy and Infectious Diseases Division of AIDS toxicity table.^24^ A protocol safety review team comprising medical and safety representatives of the sponsor and study partners and a data review committee monitored participant safety throughout the course of the trial.

Sera drawn on day 0 (baseline) and then at weeks 16, 28, 52, and 72 were used to measure humoral immune responses 4 weeks after the second, third, and fourth vaccinations, and 6 months after the fourth vaccinations. Peripheral blood mononuclear cells (PBMCs) were collected from the same blood draws and cryopreserved for analysis of antigen-specific cellular immune responses. Immunogenicity assays were performed as described in the (appendix pp 2–5).

### Statistical analysis

No prespecified statistical hypothesis was tested. The sample size was deemed appropriate to assess the safety and tolerability of the different vaccine regimens and to collect sufficient data on immunogenicity. Participants receiving placebo were included for safety evaluations and to provide control specimens for immunogenicity assays. Although mild-to-moderate vaccine reactions (local site and systemic responses) were expected, adverse events that would preclude further vaccine administration or more serious ones that would limit product development were not anticipated. Participant numbers were presecified. With 110 individuals in the tetravalent Ad26.Mos4.HIV vaccine regimen, we calculated that observing 0 such reactions would provide 95% confidence that the true rate was below 2·7%. With 165 individuals in the combined vaccine groups, we calculated that 0 reactions would provide 95% confidence that the true rate was below 1·8%. Safety analyses were done on all participants who received at least one study injection (full analysis set). Immunogenicity analyses were done on all participants who received at least the first three vaccinations according to the protocol-specified vaccination schedule (± 2 weeks), had at least one measured post-dose blood sample collected, and were not diagnosed with HIV during the study (per-protocol set). Samples taken after week 48 from participants in the per-protocol set who did not receive the fourth vaccination in the protocol-specified time window (± 2 weeks) were excluded from the analyses. No comparisons were made based on region because of the relatively small number of Rwandan participants. Analyses were done using SAS (version 9.4). Differences between the tetravalent and trivalent groups after third and fourth vaccinations were explored as geometric mean ratios (including 95% CI) with a two-sample *t* test on log-normally distributed data, or as medians by a Wilcoxon rank sum test. Anticipating a dropout rate of approximately 10%, sample sizes allowed detection of approximately 1·5-fold differences in Env-binding antibody geometric mean concentrations between Ad26.Mos4.HIV (approximately 100 evaluable participants) and Ad26.Mos.HIV (approximately 50 evaluable participants) groups with 80% probability, assuming a one-sided 5% type 1 error and an SD of 0·4 on the log_10_ scale. This study is registered with ClinicalTrials.gov, NCT02788045.

### Role of the funding source

US clinical sites were funded by the National Institute of Allergy and Infectious Diseases through the HIV Vaccine Trials Network. Funding was provided by the US Military HIV Research Program, Ragon, and Beth Israel Deaconess Medical Center. Bill & Melinda Gates Foundation provided a grant for oversight support services. The study sponsor Janssen Vaccines & Prevention funded the Rwanda site, manufactured and distributed the vaccine, and participated in data collection, data analysis, data interpretation, and writing of the report. All authors had full access to collated data. LRB and DJS led manuscript writing; all authors reviewed drafts and agreed on the final version and the decision to submit for publication. Full access to clinical and laboratory data was made available to all members of the study teams and masthead authors.

## Results

**Figure 1:**
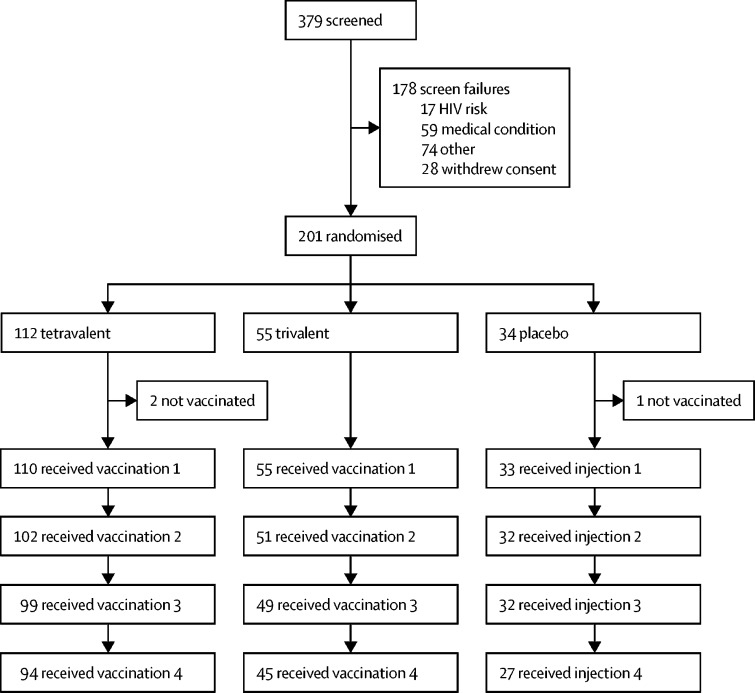
Study flowchart

Of 379 volunteers screened, 201 were enrolled and randomly assigned (figure 1). Three participants did not receive their first study injections, two in the tetravalent group and one in the placebo group. Demographic characteristics of the 198 participants who received at least one injection were similar across groups, with the exception that proportionally more women were in the placebo group. By week 72, 167 (84%) of 198 participants had completed the study and 164 (83%) had received their full series of four vaccinations (table 1). 12 (6%) of 31 study discontinuations were losses to follow-up, 11 (6%) were participant withdrawals, two (1%) were adverse events, two (1%) were pregnancies, and one (1%) was a protocol violation (table 1). Both pregnancies resulted in healthy full-term infants with no abnormalities.

**Table 1:** Demographics and baseline characteristics and of all participants who received at least one study injection

		**Tetravalent (n=110)**	**Trivalent (n=55)**	**Placebo (n=33)**
Sex				
	Female	54 (49%)	32 (58%)	24 (73%)
	Male	56 (51%)	23 (42%)	9 (27%)
Age, years		30·1 (18–50)	31·0 (19–50)	28·2 (18–44)
Country				
	USA	100 (91%)	50 (91%)	31 (94%)
	Rwanda	10 (9%)	5 (9%)	2 (6%)
Race				
	White	58 (53%)	24 (44%)	23 (70%)
	Black or African American	35 (32%)	18 (32%)	7 (21%)
	Asian	5 (5%)	5 (9%)	0
	Other	12 (11%)	8 (15%)	3 (9%)
Ethnicity				
	Hispanic or Latino	13 (12%)	6 (11%)	4 (12%)
	Not Hispanic or Latino	96 (87%)	48 (87%)	29 (88%)
	Not reported	1 (1%)	1 (2%)	0
Height, cm		170 (149–195)	170 (147–197)	169 (155–189)
Weight, kg		77 (50–150)	80 (46–141)	77 (53–132)
Body-mass index		26·3 (17·3–46·6)	27·6 (17·3–41·6)	26·9 (17·9–38·8)
Completed study				
	All visits to week 72	94 (86%)	45 (82%)	28 (85%)
	Received three vaccinations*	100 (91%)	50 (91%)	32 (97%)
	Received four vaccinations	93 (85%)	44 (80%)	27 (82%)
Reasons for study discontinuation†				
	Adverse event	0	1 (2%)	1 (3%)
	Lost to follow-up	5 (5%)	4 (7%)	3 (9%)
	Pregnancy	1 (1%)	1 (2%)	0
	Protocol violation	1 (1%)	0	0
	Withdrawal by participant	6 (6%)	4 (7%)	1 (3%)
	Other	3 (3%)	0	0

Data are n (%) or mean (min–max).

* Includes those who missed the third vaccination but received the fourth on schedule.

† Does not include the three participants who did not receive a vaccine.

**Figure 2:**
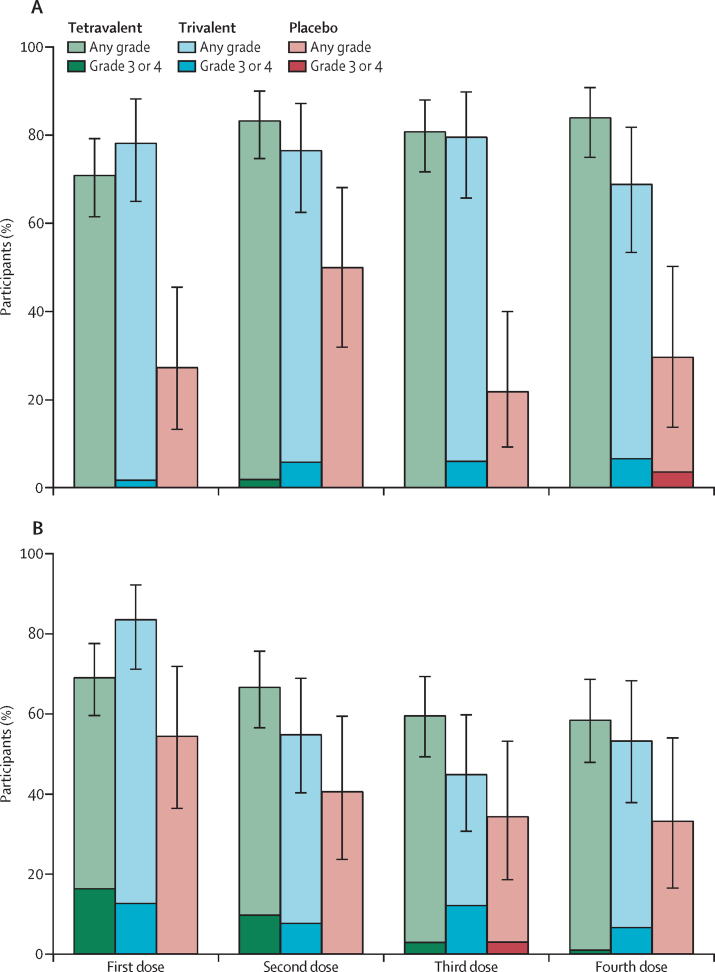
Solicited local and systemic adverse events Solicited local (A) and systemic (B) adverse events, as proportions of participants in the three study groups after each vaccination dose in the full analysis set. The full analysis set included all participants who received at least one study injection. Numbers in the columns are the total numbers of participants per group at each timepoint.

Safety data were analysed for all 198 participants who received at least one study intervention (table 2). Over all doses, solicited local adverse events were reported by 98 (89%) of 110 participants in the tetravalent group, 49 (89%) of 55 participants in the trivalent group, and 21 (64%) of 33 participants in the placebo group. Rates of solicited local adverse events were similar in the trivalent and tetravalent groups. Across the study, rates of solicited local adverse events were similar for men and women, and there was no increase with subsequent doses (figure 2A). Most local adverse events were grade 1 or grade 2 in severity. All grade 3 local adverse events were pain or tenderness, reported by two (2%) of 110 participants in the tetravalent group and six (11%) of 55 participants in the trivalent group. One (3%) placebo recipient had grade 3 erythema after the fourth injection.

**Table 2:** Summary of safety assessments in all participants who received at least one study injection

		**Tetravalent (n=110)**	**Trivalent (n=55)**	**Placebo (n=33)**
Serious adverse events				
	Any	6 (6%)	0	0
	Angina pectoris and acoustic neuroma	1 (1%)	0	0
	Atrial fibrillation	1 (1%)	0	0
	Ankle fracture	1 (1%)	0	0
	Rheumatoid arthritis	1 (1%)	0	0
	Syncope	1 (1%)	0	0
	Lymphoedema	1 (1%)	0	0
Adverse event leading to vaccine discontinuation		0	1 (2%)*	2 (6%)†
Grade 3 solicited adverse events				
	Any local	2 (2%)	6 (11%)	1 (3%)
	Any systemic	25 (23%)	13 (24%)	1 (3%)
Unsolicited adverse events within 28 days of any dose				
	Any	74 (67%)	35 (64%)	23 (70%)
	Related	5 (5%)	4 (7%)	3 (9%)
	Grade 3	2 (2%)	3 (5%)	0
Abnormal vital signs				
	Bradycardia	15 (14%)	9 (16%)	7 (21%)
	Hypertension	15 (14%)	5 (9%)	5 (15%)

Data are n (%).

* Hepatitis C infection.

† Urticaria and viral infection. There were no adverse events of special interest.

Generally, the frequency of solicited systemic adverse events decreased with subsequent vaccinations (figure 2B). The most frequent solicited systemic adverse event after any dose (>60% in any group) was fatigue, reported in 82 (75%) of 110 participants in the tetravalent group and 46 (84%) of 55 participants in the trivalent group, compared with 18 (55%) of 33 participants in the placebo group. Headache, reported by 73 (66%) of 110 participants in the tetravalent group, 34 (62%) of 55 participants in the trivalent group, and 16 (48%) of 33 participants in the placebo group, and myalgia, reported by 69 (63%) of 110 participants in the tetravalent group, 33 (60%) of 55 participants in the trivalent group, and 10 (30%) of 33 participants in the placebo group), were next most frequent in tetravalent, trivalent, and placebo groups. Pyrexia (oral temperature ≥38·0°C) was reported by 17 (15%) of 110 participants in the tetravalent group, seven (13%) of 55 participants in the trivalent group, and one (3%) of 33 participants in the placebo group, mostly after the first injections. Two cases of grade 3 pyrexia (≥39·3°C) occurred, one in the tetravalent group after the first vaccination and one in the trivalent group after the second vaccination. Grade 3 solicited systemic adverse events were more frequently reported by participants receiving the tetravalent (23%) and trivalent (24%) vaccines compared with those receiving placebo (3%). There were no grade 4 solicited systemic adverse events.

Similar frequencies of unsolicited adverse events occurred over the study duration in each group: 74 (67%) of 110 participants in the tetravalent group, 35 (64%) of 55 participants in the trivalent group, and 23 (70%) of 33 participants in the placebo group. The most frequent unsolicited adverse events were infections: 43 (39%) of 110 participants in the tetravalent group, 22 (40%) of 55 participants in the trivalent group, and 16 (49%) of 33 participants in the placebo group. Reporting rates were similar after each of the four doses. Grade 3 unsolicited adverse events were reported by five (5%) of 110 participants in the tetravalent group and three (6%) of 55 participants in the trivalent group, but in no participants in the placebo group. Six participants in the tetravalent group reported a total of seven serious adverse events: grade 2 rheumatoid arthritis and lymphoedema (occurring in one participant), grade 3 atrial fibrillation, ankle fracture, and syncope (each occurring in one participant), and grade 3 acoustic neuroma on day 93 and grade 3 angina pectoris on day 239 (occurring in one participant). The investigator considered the case of rheumatoid arthritis related to vaccination. This participant had a medical history of eczema, depression, anxiety, and multiple fractures, and developed progressive symptoms of bilateral joint pains and decreased mobility from 32 days after the final dose of tetravalent vaccine. They were diagnosed with rheumatoid arthritis about 4 months after symptoms onset. Other serious adverse events were considered unrelated to the study interventions, and no adverse events of special interest (ie, confirmed HIV infection) occurred during the study.

Laboratory abnormalities were infrequent; all were grade 1 or grade 2 except for a grade 3 increase in alanine aminotransferase concentration and a grade 3 decrease in haemoglobin concentration (each occurring in one participant in the tetravalent group). The only clinically meaningful vital sign changes were bradycardia and diastolic hypertension. Asymptomatic bradycardia was observed in 15 (14%) of 110 participants in the tetravalent group, nine (16%) of 55 participants in the trivalent group, and seven (21%) of 33 participants in the placebo group. Two cases of asymptomatic bradycardia were grade 3 (one in the tetravalent group and one in the trivalent group), and both participants already had low heart rate values at baseline. Hypertension occurred in 15 (14%) of 110 participants in the tetravalent group, five (9%) of 55 participants in the trivalent group, and five (15%) of 33 participants in the placebo group, but none of the cases were grade 3.

**Figure 3:**
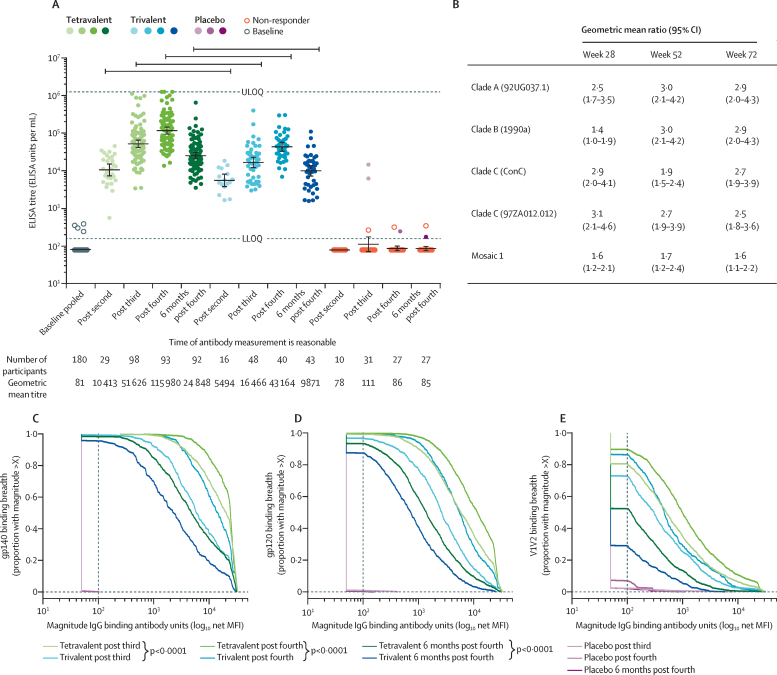
Humoral binding antibody immune responses Horizontal bars indicate matched timepoints where the response magnitude is significantly different at p<0·05. In all cases, calculated p<0·0001. Humoral binding antibody immune responses to vaccination in the per-protocol set of the three study groups at the indicated time points. The per-protocol set included all participants who received at least the first three vaccinations according to the protocol-specified vaccination schedule, had at least one measured post-dose blood sample collected, and were not diagnosed with HIV during the study. (A) ELISA responses to vaccine-matched clade C gp140 Env strain 97ZA012. (B) Comparison of tetravalent and trivalent regimens as geometric mean ratios of binding antibody responses to additional gp140 Env strains. Expanded binding antibody multiplex assay breadth panels (appendix p 11), comprising nine gp140 (C), 20 gp120 (D), and 16 gp70 V1V2 (D) antigens based on those previously described. The dotted lines indicate the lower limit of the assay. The pink lines indicate the samples from placebo recipients. ULOQ=upper limit of quantification. LLOQ=lower limit of quantification. MFI=mean fluorescence intensity.

Both active vaccine regimens were highly immunogenic but varied in the frequency and magnitude of induced immune responses throughout the study. Four (2%) of 180 participants had binding antibodies to autologous Env clade C gp140 above the lower limit of quantitation (156 EU/mL) at baseline, but an analysis of the first 55 participants after the second vaccination detected antibodies in all participants (figure 3A). After receiving only the tetravalent or trivalent Ad26 vectors encoding mosaic antigens, the geometric mean titres of clade C Env total IgG ELISA were two-fold higher in the tetravalent group (10 413 EU/mL, 95% CI 7284–14 886) than in the trivalent group (5494 EU/mL, 3759–8029). These immune responses were enhanced after the third and fourth vaccinations containing clade C gp140 protein, and the higher magnitude of response in the tetravalent group was consistent through week 72 when antibody concentrations had waned to some extent (24 848 EU/mL in tetravalent group *vs* 9871 EU/mL in trivalent group). These geometric mean ratios were significantly different when compared by two-sample *t* tests (figure 3B). Participants in the placebo group did not show any consistent responses and geometric mean titres remained below the lower limit of quantitation throughout the study.

An extended cross-clade panel of Env gp140, gp120, and gp70 V1V2 antigens established to measure the breadth of binding antibodies by binding antibody multiplex assay,^25^ and four additional Env gp140 antigens (clade A, clade B, consensus C, and Mosaic1 gp140) established to measure the breadth of binding antibodies by ELISA showed that the tetravalent group also had significantly higher concentrations of binding antibodies to breadth panels for all antigen classes, as well as to heterologous and vector-encoded Envs (figure 3C–E, appendix pp 15–18). When examined by clade C gp140 IgG1 and IgG3 subclass-specific ELISA, responses were higher in magnitude in the tetravalent group than the trivalent group throughout the study (appendix pp 16–17). IgG3 ELISA response rates were 67% in the tetravalent group compared with 18% in the trivalent group after three vaccinations, and 66% in the tetravalent group compared with 46% in the trivalent group after four vaccinations. These assays indicated greater breadth and magnitude of binding antibody with the tetravalent vaccine compared with the trivalent vaccine in all panels tested and for both IgG1 and IgG3 subtypes, despite waning of IgG3.

In addition to binding antibodies, functionality of the humoral response was measured by antibody-dependent cellular phagocytosis assay and two antibody-dependent cell-mediated cytotoxicity (ADCC) assays (appendix pp 17–19). Phagocytosis scores to the vaccine-matched clade C gp140 were significantly enhanced in the tetravalent group relative to the trivalent group, throughout the study (appendix pp 17–19). Using clade C strain TV1 in the gp120-coated target cell ADCC assay, ten (15%) of 66 participants had a response in the tetravalent group, compared with one (4%) of 27 in the trivalent group (appendix pp 17–18). Using the TV1 strain in the infectious molecular clone-infected target cell assay, response rates were proportionally higher: a 52% response rate (34 of 66 participants) in the tetravalent group and a 41% (11 of 27 participants) in the trivalent group, indicating preferential engagement of ADCC-mediating antibodies to the forms of Env present on infected cells relative to gp120-coated target cells for both vaccine groups. No neutralising responses were observed, except for easy-to-neutralise tier 1 virus MW965.26 (appendix p 20).

Cellular immune responses to vaccination were measured by IFNγ ELISPOT in response to stimulation with potential T-cell epitope or vaccine-matched peptide pools for Env, Gag, and Pol. Responses to Gag and Pol were consistent between the tetravalent and trivalent groups for all pools (appendix pp 21–26), but Env responses differed based on the vector composition. Responses to the potential T-cell epitope Env peptide pool, representing the global diversity of T-cell epitopes, were significantly higher in the tetravalent group than the trivalent group throughout the study; after the third vaccination responder rates were 96% (median 450 SFU/10^6^ PBMCs) in the tetravalent group and 79% in the trivalent group (median 255 SFU/10^6^ PBMCs). After the fourth vaccination, responder rates were 96% (median 521 SFU/10^6^ PBMCs) in the tetravalent group and 85% (median 282 SFU/10^6^ PBMCs) in the trivalent group, and at week 72 responder rates were 98% (median 397 SFU/10^6^ PBMCs) in the tetravalent group and 83% (median 243 SFU/10^6^ PBMCs) in the trivalent group (appendix pp 21–26). The vaccine-autologous mosaic peptide pools showed consistent Mos1 Env responses between vaccine groups, while the closely related Mos2 Env responses were significantly greater in the tetravalent vaccine group (appendix pp 21–26), showing that an enhanced T-cell response to one immunogen is not necessarily associated with a corresponding decrease against another.

Intracellular cytokine staining to assess the type of T cell responding to vaccination detected both CD4+ and CD8+ T cells expressing either IFNγ, IL2, or both (appendix pp 25–26). After ex vivo stimulation with Env peptide pools, the tetravalent group had an increased rate of responders throughout the study (57% *vs* 44% after three vaccinations, 76% *vs* 55% after four vaccinations, and 64% *vs* 37% at week 72 in the tetravalent and trivalent groups). The largest difference between groups was attributable to significantly higher response rates and magnitude CD4+ T-cell responses specific for the clade C gp120 (appendix pp 25–26; 0·052% *vs* 0·011% T cells and 45% *vs* 13% responders after three vaccinations; 0·074% *vs* 0·035% T cells and 62% *vs* 28% responders after four vaccinations; and 0·056% *vs* 0·017% T cells and 46% *vs* 8% responders at week 72 in the tetravalent and trivalent groups, p=0·0012), while gp41 and Mos1 gp120 responses were consistent between vaccine groups (appendix pp 25–26). CD8+ T-cell responses to any Env were similar between groups, with response rates of 42% in the tetravalent group and 46% in the trivalent group after the third vaccination, 34% in the tetravalent group and 45% in the trivalent group after the fourth vaccination, and 34% in the tetravalent group and 29% in the trivalent group at week 72 (appendix pp 27–29). T-cell responses to Pol and Gag were specifically mediated by CD8+ T cells and were similar between groups in both magnitude and frequency (appendix pp 27–29), and consistent with previously described responses for these antigens.^17^

Across all immune responses evaluated, the replacement of half of the dose of Ad26.Mos1.Env by a second mosaic Env-encoding Ad26 vector with Mosaic 2S, conferred greater overall magnitude of responses without any apparent immune interference (appendix p 29). Whether epitope-specific alterations occurred will be evaluated subsequently.

Low-to-moderate levels of pre-existing neutralising antibodies to Ad26 were present in a small proportion of study participants, primarily those from Rwanda, and were balanced across groups (appendix pp 30–31). We did not observe any effect of pre-existing, vector-specific immunity on the magnitude of immune responses induced, however only a few participants had pre-existing high titres to Ad26 (appendix pp 30–31).

## Discussion

This first-in-human phase 1/2a study of the Ad26-vectored tetravalent mosaic HIV-1 vaccine, using viral vectors with novel mosaic inserts for Env, Gag, and Pol antigens, showed increased magnitude of elicited immune responses and expansion of the breadth of humoral coverage compared with the previously tested trivalent mosaic vaccine.^17^ Including the additional Mos2S.Env component did not raise any safety concerns. Both trivalent and tetravalent vaccine regimens were generally safe and well tolerated, with acceptable reactogenicity profiles consisting of low rates of attributable severe or serious adverse events for both. These data support advancing the tetravalent vaccine into further clinical development. As previously noted with adenovirus-vectored vaccines, some local and systemic vaccination-associated reactions occur; they are typically mild, last 1 or 2 days, are more notable after the first vaccination, and diminish with subsequent doses. Overall, the safety profile we observed is consistent with previous experience,^17^, ^18^ showing consistency of the vaccine regimen safety profile across studies with largely different sites and investigative teams.

Both vaccines elicited humoral and cellular anti-HIV-1 immune responses in over 98% of vaccine recipients. Immune responses to the tetravalent vaccines were higher than the trivalent vaccine, with no evidence of any interference between the mosaic Env constructs or from anti-vector immunity from previous natural exposure. The characterisation of immunogenicity profile in this TRAVERSE study for the trivalent vaccine is consistent with the earlier APPROACH study,^17^ both in the frequency and magnitude of binding and functional antibody responses as well as cellular responses.

The tetravalent Ad26.Mos4.HIV candidate vaccine was significantly more immunogenic by several measures than the trivalent Ad26.Mos.HIV throughout the vaccination series and the follow-up period to 72 weeks after the first vaccination (appendix p 29). Increases in both humoral and cellular immune responses seen by replacing half of the Ad26.Mos1.Env dose with Ad26.Mos2S.Env support the decision to select this tetravalent vaccine for further evaluation of its ability to confer protection against HIV-1 acquisition in efficacy studies.

The coverage of circulating HIV-1 strains by the Mos1.Env is highest for clade B and CRF01_AE viruses, while the coverage by Mos2S.Env is highest for clade C strains. This complementary coverage is seen clearly in the increased magnitude of clade C gp140 binding and functional antibody responses, while in contrast with preclinical findings, further increases in the immune responses to clade A and clade B Env were also seen (appendix p 29). Similarly, the frequency and magnitude of CD4 T-cell responses to clade C gp120 peptides increased, but no corresponding attenuation of the Mos1 gp120 responses was observed. The T-cell response to potential T-cell epitope Env peptide pools increased significantly throughout the study in the tetravalent regimen group, suggesting that this regimen could induce improved immune responses relevant to the globally circulating strains of HIV (appendix p 29).

The tetravalent regimen improves the vaccine concept selected from the APPROACH and NHP studies.^17^ The studies showed that this vaccine regimen provided substantial protection against repetitive, heterologous, intrarectal SHIV-SF162P3 challenges. Furthermore, similar levels of immune response associated with protection in rhesus monkeys were also induced in humans, and support advancing this vaccine concept towards clinical efficacy testing. We have now observed that the tetravalent vaccine regimen further exceeds these same immunogenicity criteria and, despite being non-mechanistic these immunological parameters might be markers for actual protective immune responses. The principal limitation of this study is that the relevance of immune markers and vaccine protection in rhesus monkeys to clinical efficacy in humans remains to be shown. Vaccine regimen optimisation based on these markers can guide the selection of the best available candidate vaccines to advance for further testing. Future studies on how these results compare with other HIV vaccines will enable deeper insights into how the vaccine delivery system and schedules affect magnitude and quality of immune response.

The absence of neutralising antibody response detected to clinically relevant tier 2 strains, including to the vaccine-autologous strain, is consistent across studies of this vaccine concept including those where protection was achieved in non-human primates.^15^, ^17^, ^18^ Instead, the cellular and multifunctional humoral responses are reactive to diverse HIV-1 strains and have the opportunity to act against HIV virus and infected cells at multiple stages of a potential infection. These mechanisms are distinct from the presumed modes of action by which other viral vaccines confer protection. By mediating functions such as phagocytosis or cytotoxicity, the Ad26 mosaic and gp140 vaccines have the potential to be relevant for protection against diverse strains of HIV-1 that exist globally.

In conclusion, we have shown that including an additional Ad26-encoded mosaic Env increases humoral and cellular responses. The results of this study do not indicate any difference in safety and tolerability between participants receiving the tetravalent regimen and the trivalent regimen. Based in part on these results, the ongoing Imbokodo study in southern Africa (registered with ClinicalTrials.gov, NCT03060629) is measuring the protective efficacy of the tetravalent Ad26.Mos4.HIV and clade C gp140 regimen in young women. A second efficacy study (MOSAICO) for this vaccine concept using the tetravalent Ad26.Mos4.HIV is also ongoing in the Americas and Western Europe (registered with ClinicalTrials.gov, NCT03964415) in men who have sex with men and transgender individuals. Through these studies, we will learn how the immunogenicity profiles described here relate to clinical efficacy.

## Data sharing

Janssen has an agreement with the Yale Open Data Access (YODA) Project to serve as the independent review panel for evaluation of requests for clinical study reports and participant-level data from investigators and physicians for scientific research that will advance medical knowledge and public health. Data will be made available following publication and approval by YODA of any formal requests with a defined analysis plan. For more information on this process or to make a request, please visit The YODA Project site The data sharing policy of Janssen (Pharmaceutical Companies of Johnson & Johnson) is available online.
