# Supplementary material for: Safety and immunogenicity of two heterologous HIV vaccine regimens in healthy, HIV-uninfected adults (TRAVERSE): a randomised, parallel-group, placebo-controlled, double-blind, phase 1/2a study
Source: Lancet HIV. 2020 Sep 30;7(10):e688–98. doi: 10.1016/S2352-3018(20)30229-0 (PMC7529856; doi:10.1016/S2352-3018(20)30229-0)
Supplement: Supplementary appendix [file mmc1.pdf]

THE LANCET
HIV

Supplementary appendix

This appendix formed part of the original submission and has been peer reviewed. We post it as supplied by the authors.

Supplement to: Baden LR, Stieh DJ, Sarnecki M, et al. Safety and immunogenicity of two heterologous HIV vaccine regimens in healthy, HIV-uninfected adults (TRAVERSE): a randomised, parallel-group, placebo-controlled, double-blind, phase 1/2a study. *Lancet HIV* 2020; **7:** e688–98.

Supplementary material

1. Key inclusion and exclusion criteria
2. Immunogenicity methods
    a. ELISA for Total IgG Binding Antibodies to HIV Env gp140
    b. ELISA for Subclass IgG Binding Antibodies to HIV Env gp140 IgG1 & IgG3
    c. Antibody-dependent Cellular Cytotoxicity (ADCC)
        i. *Granzyme B GranToxiLux assay (ADCC-GTL)*
        ii. *Luciferase-Based Antibody-Dependent Cell-mediated Cytotoxicity Assay (ADCC-Luc)*
    d. Antibody-dependent Cellular Phagocytosis (ADCP)
    e. Neutralising Antibodies (HIV nAb; TZM-bl)
    f. Binding Antibody Multiplex Assay (BAMA) Total IgG and IgG3 Breadth
    g. Neutralising antibodies against Ad26 Vector
    h. ELISPOT for IFN-γ T Cell Responses
    i. Intracellular Cytokine Staining
3. Supplementary tables
    a. Table 1. ICS - Cytokine Response for Gag and Pol Peptide pools
    b. Table 2. BAMA panel composition
4. Supplementary figures

1. Key inclusion and exclusion criteria

Participants enrolled in this study were required to meet the following key inclusion criteria:

- Aged ≥18 to ≤50 years old on the day of signing the informed consent form.
- Healthy as determined by physical examination, medical history and vital signs measurement performed at screening.
- Had a negative HIV blood test at screening.
- Woman of childbearing potential had a negative serum pregnancy test (β-human chorionic gonadotropin) at the screening visit and a negative urine pregnancy test pre-dose on Day 1 and before each of the subsequent doses.
- A woman of childbearing potential who was heterosexually active and had a non-vasectomised partner, or partner had a non-verified or positive sperm count after a vasectomy procedure of <1 year ago, had to use an acceptable effective method of contraception.
- For heterosexually active men, contraceptive methods were dependent on child-bearing potential of the female partner with the same criteria as for female participants.
- Woman agreed not to donate eggs (ova, oocytes) for the purpose of assisted reproduction until 3 months after receiving the last dose of study vaccine/placebo. A man agreed not to donate sperm until 3 months after receiving the last study injection.
- Assessed by the clinic staff as being at low risk for HIV infection.

Participants were not to be enrolled into the study if pre-study examination revealed that they had the following exclusion criteria:

- Had chronic hepatitis B or active hepatitis C, active syphilis infection, chlamydia, gonorrhea or trichomoniasis (in female participants only). Active syphilis documented by serology unless positive serology was due to past treated infection.
- Had a history of newly acquired herpes simplex virus type 2, syphilis, gonorrhea, non-gonococcal urethritis, chlamydia, pelvic inflammatory disease, trichomoniasis, mucopurulent cervicitis, epididymitis, proctitis, lymphogranuloma venereum, chancroid or hepatitis B in the 12 months prior to randomization.
- Had any condition for which, in the opinion of the investigator, participation would not be in the best interest of the participant (e.g., compromised their well-being) or that could prevent, limit or confound the protocol-specified assessments. In case of questions, the investigator is encouraged to contact the study responsible physician.
- Had undergone major surgery within 4 weeks prior to screening or planned to undergo major surgery through the course of the study.
- Had a thyroidectomy or active thyroid disease requiring medication during the last 12 months (not excluded: a stable thyroid supplementation).

2. Immunogenicity Methods

a. ELISA for Total IgG Binding Antibodies to HIV Env gp140

Vaccine-induced binding antibody (Ab) responses were investigated using several HIV-1 Env Clades/strain specific enzyme-linked immunosorbent assay (ELISAs). The assay was validated and was performed at Janssen Vaccines & Prevention B.V., Leiden, the Netherlands.

Antibody binding to five different gp140 antigens was determined by ELISA. The gp140 antigens that were used are Clade A (92UG037.1), Clade B (1990a), Clade C (Con C), Clade C (C97ZA.012) and Mos1. The HIV Env antigen of interest was coated on 96-well microtiter plates. Serum samples were added to the plates and HIV Env-specific Abs were detected with a mouse anti-human immunoglobulin G (IgG) Ab conjugated with horseradish peroxidase (HRP) followed by a colorimetric reaction (tetramethylbenzidine [TMB] substrate). Each plate contained a 12-point reference standard and a high, medium and low negative control. Four-parameter logistic regression was used to fit the reference curve. All samples, the reference standard and the controls were measured in duplicate. The highest concentration of the reference standard was arbitrarily set at 100.000 ELISA units per mL (EU/mL).

The lower limits of quantification (LLOQs) taken as reference for response to this assay were 625, 156.25, 625, 156.25 and 78.125 EU/mL for Clade A (92UG037.1), Clade B (1990a), Clade C (Con C), Clade C (C97ZA.012) and Mos1, respectively.

b. ELISA for Subclass IgG Binding Antibodies to HIV Env gp140 IgG1 & IgG3

Vaccine-induced binding Ab IgG1 and IgG3 subclass responses were investigated using Clade C (C97ZA.012) specific ELISAs. The assay was qualified and was performed at the BIDMC, Boston, MA, USA.

Plates were coated with 150 ng/well Clade C (C97ZA.012) gp140 protein overnight at 4°C. After this overnight incubation, the plates were washed with ELISA wash and were blocked for 3-4 hours at room temperature. All steps following the blocking step were done at room temperature. Serum was added in duplicate, serial diluted 2-fold and then allowed to incubate for 1 hour. Plates were washed and HRP-conjugated anti-human IgG1 or IgG3 secondary Ab was added to each well at the appropriate dilution and incubated for 1 hour. Plates were washed again, SureBlue was added to each well for 10 minutes followed by TMB stop solution. Plates were then read at 450 nm. A 4-parameter logistical curve fit was used to calculate the 50% effective concentration (EC_{50}) titer for each sample.

The LLOQs for this assay were 12.3 and 12.4, for IgG1 and IgG3, respectively.

c. Antibody-dependent Cellular Cytotoxicity (ADCC)

i. Granzyme B GranToxiLux assay (ADCC-GTL)

The ADCC-GranToxiLux (ADCC-GTL) assay is based on the hydrolysis of a cell-permeable fluorogenic peptide substrate containing a sequence recognised by the serine protease, Granzyme B (GzB).[1] Granzyme B is delivered into target cells by cytotoxic effector cells as a result of antigen (Ag)-specific Ab-Fc-gamma receptor interactions. Within the target cells, effector cell-derived GzB hydrolyzes the substrate, generating a fluorescent signal that allows individual cells in which cell death has been triggered by effector cells to be identified by flow cytometry. The ADCC-GTL assay was developed at Duke University, NC, USA.

The assay utilizes a flow-cytometry-based detection system with gp120 (TV-1) or gp140 (C97ZA.012 or Mos1)-coated target cells (CEM Natural Killer Resistant Cell line (CEM.NKR.CCR5)) that may recapitulate the gp120 or gp140 conformation at time of virus entry and, in general, upon binding to the CD4 molecule on the surface of the T-cells. In this assay, the target cells are labeled with a fluorescent peptide representing a specific substrate for GzB and a viability dye that allows for discrimination of target cells that were dead before incubation with serially diluted serum and effector cells at an effector to target ratio of 30:1 (thawed PBMCs that are obtained from cryopreserved leukapheresis samples). Upon recognition of the target cells, mediated by the anti-Env Ab-Fc Receptor (Ab-FcR) interaction, the GzB is delivered by the effector cells into the target cells, where it then cleaves the fluorescent peptide and releases a fluorescent signal that can be identified by flow cytometry. The final read-out is the frequency (percentage) of GzB positive cells per serum dilution and results are reported as percentage GzB activity (%GzB) as well as the area under the curve (AUC) %GzB of the respective serum dilutions.

The LLOQ was not determined for the assay, however a positivity threshold of 8% GzB was established, based on seronegative participants.

1. Pollara J, Hart L, Brewer F, et al. High-throughput quantitative analysis of HIV-1 and SIV-specific ADCC-mediating antibody responses. Cytometry A 2011;79A:603–612.

ii. Luciferase-Based Antibody-Dependent Cell-mediated Cytotoxicity Assay (ADCC-Luc)

The Luciferase-Based Antibody-Dependent Cell-mediated Cytotoxicity Assay (ADCC-Luc) assay is based on HIV Infectious Molecular clone (IMC) viruses expressing the Renilla luciferase (LucR) reporter gene that are used to infect the target cells. The effector cell populations are whole PBMCs that are obtained from cryopreserved leukapheresis samples. In this assay, the serum samples are incubated with the target cells in presence of the effector cells for 6 hours. The final read-out is the reduction in luminescence intensity generated by the residual intact target cells that have not been lysed by the effector population in presence of ADCC-mediating antibodies. The ADCC-Luc assay was developed at Duke University, NC, USA.

The assay procedure is as follows: Target cells (CEM.NKR.CCR5 cell line) infected with the HIV-1 IMC-LucR (TV-1 or MW96.5) are combined with effector cells (thawed PBMCs incubated with IL-15 during an overnight

rest) in the presence of serially diluted serum, followed by a 6 hours incubation at 37ºC and 5% CO_2. Luciferase activity is then measured using the Promega Vivi-Ren assay.

Percentage killing is calculated as follows: (Relative Light Units [RLU] of target and effector cell well) – (RLU of test well: serum, target and effector cells) / (RLU of target and effector cell well) x 100. Results for post-vaccination samples are reported as the % killing after matched baseline-subtracted % killing per serum dilution, as well as the partial Area under the Baseline-subtracted Curve (pABC) of the 4 lowest serum dilutions of the curve (1:50, 1:200, 1:800, 1:3200).

The LLOQ was not determined for the assay, however a positivity threshold of 10% baseline-subtracted killing was established. For a post-baseline sample to be considered a positive vaccine response, one of the first two dilutions (1:50 and 1:200) must be above the 10% positivity threshold.

d. Antibody-dependent Cellular Phagocytosis (ADCP)

The functionality of vaccine-induced Ab responses was investigated by the determination of ADCP. The assay was qualified and was performed at the Ragon Institute, Boston, MA, USA as previously described.[1]

The ADCP assay was used to evaluate the ability of antibodies to drive antigen-uptake following immune complex formation by effector cells like macrophages, neutrophils or dendritic cells. Fluorescent, streptavidin-coated beads were labelled with biotinylated Env proteins of Clade A (92UG037.1), Clade B (1990a), Clade C (Con C), Clade C (C97ZA.012) and Mos1. These antigen-coated beads were then incubated with 1:100 diluted clinical sample and the Ab-opsonised beads were then added to human monocytic THP-1 cells. The extent of ADCP activity was determined by flow cytometry. The percentage of bead positive THP-1 cells (representing the number of THP-1 cells that took up beads) and the mean fluorescent intensity (MFI, representing the number of beads taken up by positive cells) were calculated and expressed as phagocytic score. The phagocytic score was defined as: (% bead positive cells) × (MFI of beads positive cells/10,000). Each assay contained one negative and one positive quality control serum sample to guarantee assay validity.

The lower limits of detection for this assay were 5.16, 6.43, 6.49, 4.32 and 4.28 (phagocytic score) for Clade A (92UG037.1), Clade B (1990a), Clade C (Con C), Clade C (C97ZA.012) and Mos1, respectively.

1. Ackerman ME, Moldt B, Wyatt RT, et al. A robust, high-throughput assay to determine the phagocytic activity of clinical antibody samples. J Immunol Methods 2011;7;366(1-2):8-19.

e. Neutralising Antibodies (HIV nAb; TZM-bl)

The functionality of vaccine-induced Ab responses was investigated by the determination of neutralising antibody (nAb) activity in a virus neutralization assay (VNA) using TZM-bl cells and Env-pseudotyped viruses at the Duke University, Durham, NC, USA. The nAb (TZM-bl) assay was developed with a grant of HVTN by Dr. Montefiori and colleagues, Duke University and was validated at Duke University.[1]

Neutralising antibodies were measured in TZM-bl cells as a function of reduction in Tat-induced Firefly Luciferase (Luc) reporter gene expression after a single round of infection with molecularly cloned Env-pseudotyped viruses.[2] For this study, viruses with a Tier 1 neutralization phenotype: Clade C: MW965.26 and 97ZA012.12 and Tier 2 neutralization phenotype: Clade C: ZM233M, CE703010010, 2759058, ZM215F, SO431, CE704810053[10] were used. A fixed amount of pseudoviruses was mixed with a serially diluted clinical serum sample. After 1-hour incubation, TZM-bl cells were added to the mixture. Pseudovirus infection or inhibition was measured at 48 hours by the Luc reporter gene expression system. Assay responses of serially diluted sample were plotted in a four-parameter logistic regression curve and the 50% inhibitory concentration (IC_{50}) was reported as the neutralization titer. The LLOQ for this assay is an IC_{50} of 10 (fold-dilution).

1. Sarzotti-Kelsoe M, Daniell X, Todd CA, et al. Optimization and validation of a neutralizing antibody assay for HIV-1 in A3R5 cells. J Immunol Methods 2014;409:147–160.
2. Montefiori DC. Evaluating neutralizing antibodies against HIV, SIV and SHIV in luciferase reporter gene assays. Curr Protoc Immunol 2005 Jan;Chapter 12:Unit 12.11.

f. Binding Antibody Multiplex Assay (BAMA) Total IgG and IgG3 Breadth

The HIV-1 Binding Antibody Multiplex Assay (BAMA) employs flow-cytometric-based technology that also utilizes Ab and antigen interactions to test for the presence of specific antibodies in an unknown sample with the added advantage of multiplexing the antigens of interest. The BAMA assay was developed and validated at Duke University, NC, USA.[1] Briefly, partially purified HIV antigens were covalently coupled to specific fluorescent bead sets. Bead sets coupled to antigens of interest were mixed together to allow for multiplexing and samples were added to the beads. This step was followed by the addition of a secondary anti-human Ab conjugated to biotin and/or phycoerythrin (PE) to allow for a fluorescent readout of secondary Ab binding. Positive and negative control standards were run with each assay to ensure specificity. The HIV-BAMA was provided to determine Ab isotype (IgG) and subclass responses (IgG3) to a broad panel of HIV-1 Env for magnitude and breadth as determined by a global antigen panel[1] against gp120, gp140, gp41 and gp70 (V1V2) adapted to this study; Supplementary Table 1 contains details on the antigen panel composition. Area under the magnitude-breadth calculations was determined as previously described and utilized.[2,3]

The positivity threshold was determined per antigen based on the +3 standard deviation (SD) on the non-specific background. Sample values had to be greater than or equal to this value and had to be 3-fold over the baseline values with a minimum MFI value of 100.

1. Yates NL, deCamp AC, Korber BT, et al. HIV-1 Envelope Glycoproteins from diverse clades differentiate antibody responses and durability among vaccinees. J Virol 2018; 92:1–20.
2. Huang Y, Gilbert PB, Montefiori DC, et al. Simultaneous evaluation of the magnitude and breadth of a left and right censored multivariate response, with application to HIV vaccine development. Stat Biopharm Res 2009; 1:81–91.
3. Van der Meeren O, Jongert E, Seaton KE, et al. Persistence of vaccine-elicited immune response up to 14 years post-HIV gp120-NefTat/AS01B vaccination. Vaccine 2020; 38:1678–89.

Table 1: **Intracellular cytokine staining - Cytokine Response for Gag and Pol Peptide pools - Descriptive Statistics of the Actual Values over Time**

	Tetravalent	Trivalent	Placebo
**CD4+**			
HIV Gag (Mos1) IFNγ+ or IL2+ (%)			
**Week 28**			
N	97	48	31
Median	0.017651	0.015194	0.000100
IQ range	0.004793-0.029686	0.007426-0.024568	0.000100-0.001332
Min, Max	0.000100, 0.115295	0.000100, 0.146626	0.000100, 0.016075
Responders n (%) (95% CI)	8 (8%) (3.63;15.61)	3 (6%) (1.31;17.20)	0
Median of Responders	0.064214	0.044755	
**Week 52**			
N	91	40	27
Median	0.016829	0.011742	0.000100
IQ range	0.004598-0.025284	0.001526-0.021820	0.000100-0.003123
Min, Max	0.000100, 0.126744	0.000043, 0.104907	0.000100, 0.024998
Responders n (%) (95% CI)	3 (3%) (0.69;9.33)	4 (10%) (2.79;23.66)	0
Median of Responders	0.061579	0.074174	
**Week 72**			
N	78	38	25
Median	0.009086	0.003595	0.000100
IQ range	0.002969-0.027008	0.000209-0.014215	0.000100-0.001733
Min, Max	0.000100, 0.067128	0.000100, 0.116649	0.000100, 0.011286
Responders n (%) (95% CI)	2 (3%) (0.31;8.96)	2 (5%) (0.64;17.75)	0
Median of Responders	0.049427	0.099338	
HIV Pol RNAseInt 1 (Mos1) IFNγ+ or IL2+ (%)			
**Week 28**			
N	96	48	31
Median	0.015104	0.013724	0.000100
IQ range	0.005574-0.027478	0.006106-0.023351	0.000100-0.004004
Min, Max	0.000043, 0.072633	0.000100, 0.151341	0.000100, 0.012014
Responders n (%) (95% CI)	6 (6%) (2.33;13.11)	1 (2%) (0.05;11.07)	0
Median of Responders	0.051563	0.151341	
**Week 52**			
N	91	40	27
Median	0.014281	0.016082	0.000259
IQ range	0.004691-0.024368	0.005344-0.028088	0.000100-0.007673
Min, Max	0.000100, 0.074572	0.000100, 0.119321	0.000100, 0.017679

Responders n (%) (95% CI)	3 (3%) (0.69;9.33)	1 (3%) (0.06;13.16)	0
Median of Responders	0.055456	0.119321	
**Week 72**			
N	78	38	25
Median	0.011639	0.010532	0.000113
IQ range	0.003491-0.027780	0.000452-0.022991	0.000100-0.005838
Min, Max	0.000100, 0.069663	0.000100, 0.146818	0.000100, 0.017382
Responders n (%) (95% CI)	3 (34%) (0.80;10.83)	2 (5 %) (0.64;17.75)	0
Median of Responders	0.068300	0.109826	
Responders n (%) (95% CI)	4 (4%) (1.15;10.33)	1 (2%) (0.05;11.07)	
Median of Responders	0.011290	0.081993	
HIV Pol RT (Mos1) IFNγ+ or IL2+ (%)			
**Week 28**			
N	97	48	31
Median	0.012602	0.016704	0.000100
IQ range	0.001563-0.022553	0.006137-0.029505	0.000100-0.005126
Min, Max	0.000036, 0.126202	0.000100, 0.070964	0.000100, 0.029891
Responders n (%) (95% CI)	4 (4%) (1.13;10.22)	4 (8%) (2.32;19.98)	0
Median of Responders	0.061006	0.050390	
**Week 52**			
N	91	40	27
Median	0.012605	0.019080	0.000100
IQ range	0.004382-0.022514	0.008738-0.030495	0.000100-0.004550
Min, Max	0.000100, 0.093141	0.000100, 0.107906	0.000100, 0.018185
Responders n (%) (95% CI)	4 (4%) (1.21;10.87)	3 (8%) (1.57;20.39)	0
Median of Responders	0.070389	0.047934	
**Week 72**			
N	78	38	25
Median	0.010277	0.012506	0.000972
IQ range	0.000100-0.018855	0.000100-0.018261	0.000100-0.002281
Min, Max	0.000100, 0.084783	0.000100, 0.090781	0.000100, 0.007861
Responders n (%) (95% CI)	3 (4%) (0.80;10.83)	2 (5%) (0.64;17.75)	0
Median of Responders	0.057013	0.078052	
**CD8+**			
HIV Gag (Mos1) IFNγ+ or IL2+ (%)			
**Week 28**			
N	96	48	31

Median	0.029117	0.036459	0.000305
IQ range	0.003776-0.313271	0.008120-0.203260	0.000100-0.004932
Min, Max	0.000069, 7.246449	0.000100, 4.086821	0.000100, 1.496572
Responders n (%) (95% CI)	38 (40%) (29.75;50.08)	19 (40%) (25.77;54.73)	1 (3%) (0.08;16.70)
Median of Responders	0.413615	0.287083	1.496572
**Week 52**			
N	91	40	27
Median	0.022546	0.034027	0.000100
IQ range	0.002438-0.183517	0.001802-0.167995	0.000100-0.004126
Min, Max	0.000100, 7.033542	0.000068, 4.956864	0.000029, 3.994355
Responders n (%) (95% CI)	29 (32%) (22.49;42.47)	13 (33%) (18.57;49.13)	1 (4%) (0.09;18.97)
Median of Responders	0.383814	0.434693	3.994355
**Week 72**			
N	79	38	25
Median	0.026651	0.034605	0.000100
IQ range	0.000100-0.166625	0.005222-0.113990	0.000100-0.001415
Min, Max	0.000100, 4.593903	0.000050, 4.069145	0.000026, 3.154841
Responders n (%) (95% CI)	26 (33%) (22.75;44.40)	12 (32%) (17.50;48.65)	1 (4%) (0.10;20.35)
Median of Responders	0.302746	0.244090	3.154841
HIV Pol RNAseInt 1 (Mos1) IFNγ+ or IL2+ (%)			
**Week 28**			
N	95	48	31
Median	0.052354	0.056631	0.000100
IQ range	0.010695-0.258820	0.011676-0.161994	0.000100-0.002495
Min, Max	0.000100, 5.083424	0.000064, 1.966761	0.000100, 0.060001
Responders n (%) (95% CI)	45 (47%) (37.03;57.88)	23 (48%) (33.29;62.81)	0
Median of Responders	0.265439	0.164864	
**Week 52**			
N	91	40	27
Median	0.032884	0.050373	0.000100
IQ range	0.004938-0.141887	0.006455-0.118912	0.000100-0.003700
Min, Max	0.000100, 5.150197	0.000100, 0.896690	0.000100, 0.007117
Responders n (%) (95% CI)	35 (38%) (28.45;49.25)	14 (35%) (20.63;51.68)	0
Median of Responders	0.313140	0.180602	
**Week 72**			
N	79	38	25
Median	0.027028	0.024823	0.000352
IQ range	0.003463-0.129852	0.003928-0.074236	0.000100-0.002950
Min, Max	0.000100, 3.900521	0.000047, 0.870954	0.000058, 0.021506

Responders n (%) (95% CI)	27 (34%) (23.87;45.71)	11 (29%) (15.42;45.90)	0
Median of Responders	0.259613	0.143950	
HIV Pol RT (Mos1) IFNγ+ or IL2+ (%)			
**Week 28**			
N	96	48	31
Median	0.025532	0.040750	0.000100
IQ range	0.001379-0.170292	0.007816-0.104330	0.000100-0.002554
Min, Max	0.000100, 2.220115	0.000100, 2.909738	0.000100, 0.016694
Responders n (%) (95% CI)	40 (42%) (31.68;52.18)	19 (40%) (25.77;54.73)	0
Median of Responders	0.219674	0.154624	
**Week 52**			
N	91	40	27
Median	0.023775	0.043065	0.000182
IQ range	0.004990-0.128189	0.005132-0.070263	0.000100-0.003168
Min, Max	0.000100, 2.777000	0.000100, 2.159989	0.000100, 0.046613
Responders n (%) (95% CI)	32 (35%) (25.44;45.88)	10 (25%) (12.69;41.20)	0
Median of Responders	0.192599	0.338342	
**Week 72**			
N	79	38	25
Median	0.028252	0.018767	0.000100
IQ range	0.003772-0.135424	0.000194-0.048483	0.000100-0.000100
Min, Max	0.000024, 1.751891	0.000100, 1.870269	0.000100, 0.013381
Responders n (%) (95% CI)	26 (33%) (22.75;44.40)	7 (18%) (7.74;34.33)	0
Median of Responders	0.183335	0.225631	

**Table 2. Binding antibody multiplex assays panel composition**	
**Antigen Class**	**Antigen Name**
**IgG-t Extended Clade C gp120**	HIV ENV gp120 Clade C (1394C9) IgG-t Ab
	HIV ENV gp120 Clade C (1428) IgG-t Ab
	HIV ENV gp120 Clade C (1641A7) IgG-t Ab
	HIV ENV gp120 Clade C (96ZM651) IgG-t Ab
	HIV ENV gp120 Clade C (CAP210) IgG-t Ab
	HIV ENV gp120 Clade C (CAP45) IgG-t Ab
	HIV ENV gp120 Clade C (CH505TF) IgG-t Ab
	HIV ENV gp120 Clade C (Ce0042) IgG-t Ab
	HIV ENV gp120 Clade C (Du156) IgG-t Ab
	HIV ENV gp120 Clade C (TV1c8) IgG-t Ab
**IgG-t gp120**	HIV ENV gp120 Clade A (51802) IgG-t Ab
	HIV ENV gp120 Clade AE (254008) IgG-t Ab
	HIV ENV gp120 Clade AE (A244) IgG-t Ab
	HIV ENV gp120 Clade B (B.6240) IgG-t Ab
	HIV ENV gp120 Clade B (BORI) IgG-t Ab
	HIV ENV gp120 Clade B (TT31P) IgG-t Ab
	HIV ENV gp120 Clade BC (CNE20) IgG-t Ab
	HIV ENV gp120 Clade BC(BJOX002) IgG-t Ab
	HIV ENV gp120 Clade C(1086C_D7) IgG-t Ab*
	HIV ENV gp120/B Clade M (Con 6) IgG-t Ab*
**IgG-t gp140**	HIV ENV gp140 Clade B (SC42261) IgG-t Ab
	HIV ENV gp140 Clade C (CH505TF) IgG-t Ab
	HIV ENV gp140C Clade A (9004S) IgG-t Ab
	HIV ENV gp140C Clade B(RHPA4259)IgG-t Ab
	HIV ENV gp140C Clade B(WITO4160)IgG-t Ab
	HIV ENV gp140C Clade C (1086C) IgG-t Ab
	HIV ENV gp140C Clade C (BF1266) IgG-t Ab
	HIV ENV gp140CF CladeAE (conAE) IgG-t Ab
	HIV ENV gp140CFI Clade M(Con S) IgG-t Ab*
**IgG-t gp70**	HIV ENV gp70 Clade A (191084)IgG-t Ab
	HIV ENV gp70 Clade AE (C2101) IgG-t Ab
	HIV ENV gp70 Clade AE (CM244) IgG-t Ab
	HIV ENV gp70 Clade B (62357.14) IgG-t Ab
	HIV ENV gp70 Clade B (CaseA) IgG-t Ab
	HIV ENV gp70 Clade B (RHPA4259) IgG-t Ab
	HIV ENV gp70 Clade B (TT31P) IgG-t Ab
	HIV ENV gp70 Clade B(700010058) IgG-t Ab
	HIV ENV gp70 Clade BC (BJOX) IgG-t Ab
	HIV ENV gp70 Clade C (96ZM651) IgG-t Ab
	HIV ENV gp70 Clade C (BF1266) IgG-t Ab
	HIV ENV gp70 Clade C (CAP210) IgG-t Ab
	HIV ENV gp70 Clade C(Ce1086) IgG-t Ab
	HIV ENV gp70 Clade C(TV1.21)IgG-t Ab
	HIV ENV gp70 CladeC(001428)IgG-t Ab
	HIV ENV gp70 CladeC(7060101641)IgG-t Ab

* These antigens are study-specific additions to the previously defined breadth panels.

g. Neutralising antibodies against Ad26 Vector

Immune responses directed against Ad26 were evaluated with an adeno-VNA. The assay was developed and validated at Janssen Vaccines and Prevention B.V., Leiden, the Netherlands.

Ad26 nAb were measured, as adapted from Sprangers et al.[1] The Ad26 VNA detects the amount of Ad26 neutralising antibodies present in serum samples, which can inhibit the infection of A549 cells by Ad26. To this end, human serum samples were titrated in 2-fold steps, after which virus which contains the reporter gene for Luc (Ad26.luc), were added. After 30 to 90 minutes incubation, cells were added to the neutralization mix and further incubated for 24 hours. Subsequently, Luc substrate (NeoLite) was added which lyses the cells and functional expression of the reporter gene by Ad26 is measured. As a control, serum samples with a defined 90% inhibitory concentration (IC90) values were included in each run. The curves were plotted and from a four-parameter logistic regression curve the IC90 was calculated and reported as the neutralization titer.

The assay LLOQ is an IC90 of 17.

1. Sprangers MC, Lakhai W, Koudstaal W, et al. Quantifying adenovirus-neutralizing antibodies by luciferase transgene detection: addressing preexisting immunity to vaccine and gene therapy vectors. J Clin Microbiol 2003; 41:5046–5052.

h. ELISPOT for IFN-γ T-Cell Responses

Frozen PBMCs were analysed by IFNγ enzyme-linked immunospot assay (ELISPOT) at the BIDMC, Boston, MA, USA. The assay was validated at Merck laboratories and successfully transferred to BIDMC as concordance was demonstrated between the laboratories.

Peripheral blood mononuclear cells were stimulated with peptides pools (15 amino acid peptides overlapping by 11 amino acids, 2 mcg per peptide/mL with 2×10^5 cells/well) matched to the vaccine inserts of the Mos1, Mos2 constructs used for vaccination and potential T-cell epitopes (PTE) of the Env, Pol and Gag proteins. The secretion of IFNγ by the peptide-stimulated PBMCs was performed with commercially available reagents[8]. The number of spot-forming cells (SFCs) per 10^6 stimulated PBMCs, after subtraction of mock stimulated PBMCs (dimethyl sulfoxide [DMSO] only) for a complete peptide pool, was reported. In each assay, only clinical PBMC samples were stimulated. A clinical sample was considered valid when mock stimulation of PBMC with DMSO resulted in <35 SFC/10^6 PBMCs and a non-specific inducer (ConA) resulted in a response >10 SFC/10^6 PBMCs.

The threshold for the ELISPOT test was based on the 95th percentile from the baseline values of the larger HIV-V-A004 study.[1]

1. Barouch DH, Tomaka FL, Wegmann F, et al. Evaluation of a mosaic HIV-1 vaccine in a multicentre, randomised, double-blind, placebo-controlled, phase 1/2a clinical trial (APPROACH) and in rhesus monkeys (NHP 13-19). Lancet 2018; 392:232–43.

i. Intracellular Cytokine Staining

To further characterise the antigen-specific T-cell responses, intracellular cytokine staining (ICS) was performed at Fred Hutchinson Cancer Research Center, HVTN, Seattle, Washington, USA. The assay is considered validated for $CD4^+$ and $CD8^+$ T-cells with the following effector functions: IFNγ and/or interleukin-2 (IL-2), other markers include: viability dye, CD14, CD45RA, CCR7, CD154, Granzyme, IL-17, IL-4, tumor necrosis factor alpha (TNFα) CD56, CXCR5, PD-1 and inducible co-stimulatory (ICOS) molecule.

A modified 17-marker panel[1] was used in the ICS assay to measure the proportion of $CD4^+$ and $CD8^+$ T-cells producing IFNγ and/or IL-2 (and other exploratory markers listed above) after stimulation of the PBMCs with vaccine-matched peptide pools to the Mosaic 1 inserts (RT, RNAse/Integrase, Gag, gp120 and gp41), and to the recombinant gp140 protein, Env Clade C (C97ZA.012) (gp120, gp41). Cryopreserved PBMCs were thawed and rested overnight, and then stimulated with HIV peptide pools for 6 hours in the presence of brefeldin A and monensin to block cytokine secretion. After the incubation, a cell surface staining was performed for viability, CXCR5, PD-1, ICOS, CD45RA, CCR7, CD56, and CD14. Subsequently, cells were fixed and permeabilised, after which an intracellular staining for cytokines was performed. Results were reported as the percentage of $CD4^+$ or $CD8^+$ T-cells with the different effector functions as measured by flow cytometry. In each assay, clinical PBMC samples were stimulated. A clinical sample was considered valid when it met the acceptance criteria for viability, cell recovery, and background level expression of IFNγ and/or IL-2 in the negative control stimulations.

Positivity criteria were set with two-by-two contingency tables derived for each comparison between the stimulated and unstimulated, cytokine-positive and negative data for each cytokine subset. The 4 entries in the table were the number of cells positive for the cytokine subset and the number of cells negative for the cytokine subset, for both the stimulated and the negative control data. A one-sided Fisher's Exact Test was applied to each table, testing whether the number of cytokine-producing cells for the stimulated data was greater than that for the negative control data. The adjusted p-values were used to determine positivity, with values less than or equal to $\alpha=10^{-5}$ indicating a positive response.

1. Moncunill G, Dobaño C, McElrath MJ, De Rosa SC. OMIP-025: Evaluation of Human T-And NK-cell Responses Including Memory and Follicular Helper Phenotype by Intracellular Cytokine Staining. Cytometry A. 2015;87:289–92.

Supplementary figure 1

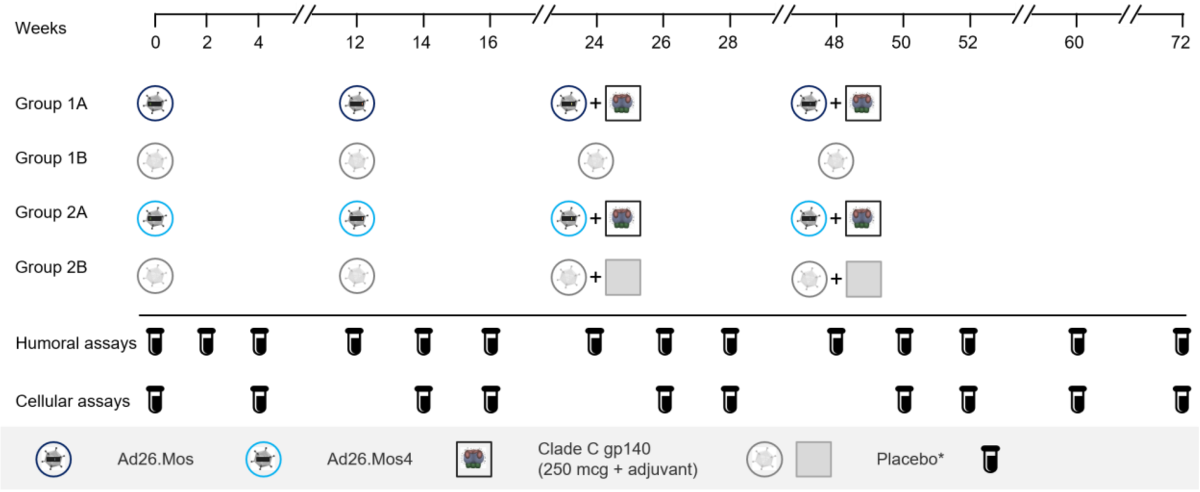

Participants received a first injection of tetravalent vaccine, Ad26.Mos4.HIV or placebo, or trivalent vaccine, Ad26.Mos.HIV, or placebo, on Day 0 and those injections were repeated 12 weeks later. At Week 24, vaccine groups received a third dose of tetravalent or trivalent together with clade C gp140, and this was repeated at Week 48, with placebos again administered to the placebo group.

Supplementary figure 2

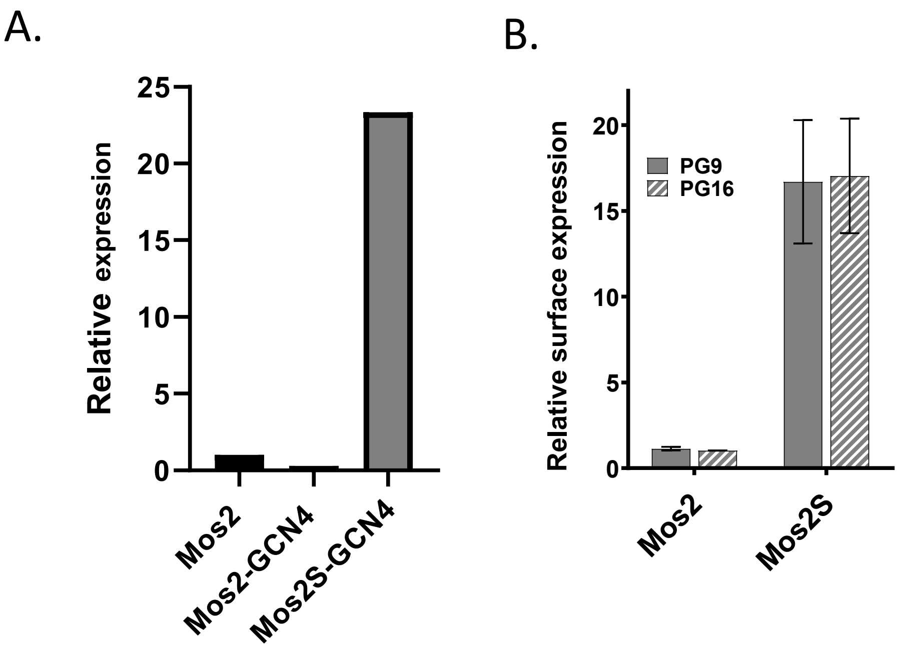

(A). Relative expression levels of soluble Mos2 HIV envelope protein (set at 1), Mos2 with a C-terminal GCN4 domain and the hybrid Mos2S with a C-terminal GCN4 domain. Expression is based on arbitrary intensity units as measured by quantitative Western blot using a polyclonal antibody against gp120. (B) Relative cell surface expression levels of the membrane-bound HIV Env after transient transfection with a plasmid coding for the membrane anchored Mos2 and hybrid Mos2S design. Env expression on transfected cells was measured by FACS analysis broadly neutralizing antibodies PG9 and PG16. Surface expression is shown relative to gfp (green fluorescent protein) negative control.

Supplementary figure 3

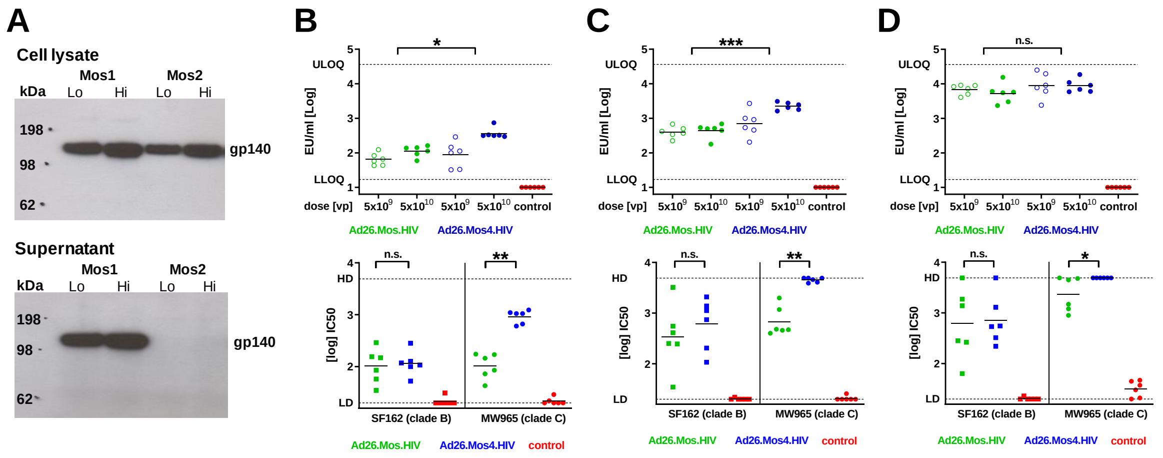

Nonclinical assessment of Mos2 Env transgene expression and immunogenicity. (A) Western blot analysis of cells transduced with the original vectors Ad26.Mos1.Env (left two lanes) and Ad26.Mos2S.Env (right two lanes); (B–D) Immunogenicity of Ad26.Mos4.HIV (including the novel Ad26.Mos2S.Env vector) compared to Ad26.Mos.HIV in rabbits. New Zealand White rabbits were immunised at week 0 and week 6 with the two Ad26 vector combinations at two dose levels ($5x10^9$ vp and $5x10^{10}$ vp; n=6/group) and at weeks 12 and 18 with 10 µg of the recombinant HIV Env protein Clade C gp140 in aluminium phosphate adjuvant. Immunogenicity was determined by Clade C ELISA (top) or by TZM-bl neutralization assays (bottom) using the pseudoviruses SF162 (Tier 1, Clade B) or MW965 (Tier 1, Clade C). The in-life phase of the study was conducted at Covance Research Products (Denver, PA, USA) and was approved by the Covance Institutional Animal Care and Use Committee (IACUC). All procedures in this study were conducted in compliance with the U.S. Department of Agriculture's (USDA) Animal Welfare Act (9 CFR Parts 1, 2, and 3); the Guide for the Care and Use of Laboratory Animals (Institute of Laboratory Animal Resources, National Academy Press, Washington, D.C., 2011); and the National Institutes of Health, Office of Laboratory Animal Welfare. Whenever possible, procedures in this study were designed to avoid or minimize discomfort, distress, and pain to animals. Open symbols: low Ad26 vector dose ($5x10^9$ vp); filled symbols: high Ad26 vector dose ($5x10^{10}$ vp). ULOQ, upper limit of quantitation; LLOQ, lower limit of quantitation; HD, highest tested dilution; LD, lowest tested dilution; *$p<0.05$, **$p<0.01$, ***$p<0.001$; n.s., not significant.

Supplementary figure 4

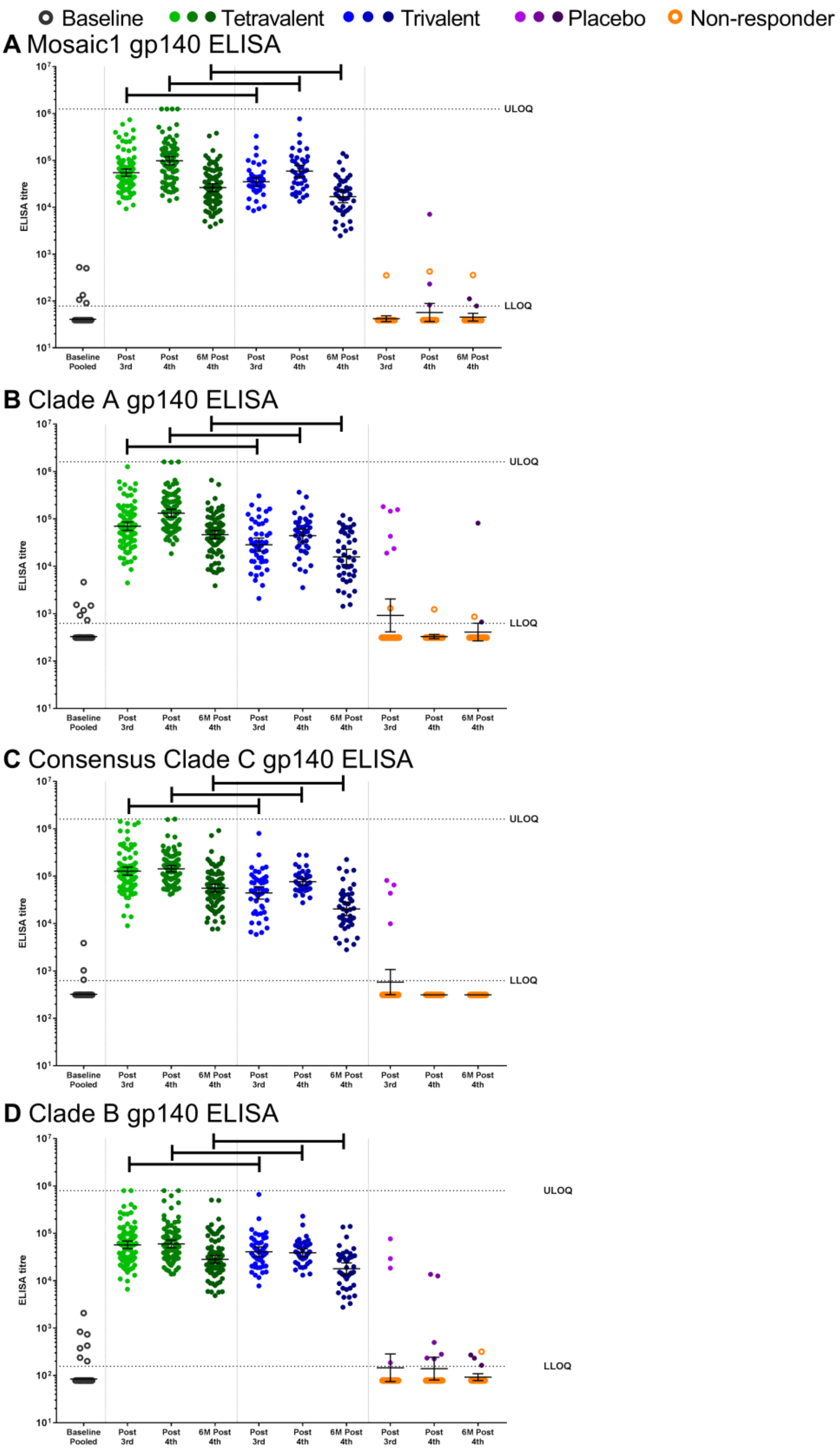

Humoral binding antibody immune responses to vaccination in humans. Response rates are shown for each vaccine group at baseline, after the third vaccination at weeks 28, after fourth vaccination at week 52 and 24 weeks after the fourth vaccination at week 72. gp140 Env ELISA responses for (A) Mosaic1 gp140, (B) Clade A 92UG037.1 (C) Consensus Clade C (D) Clade B 1990a. The dotted lines are the LLOQ and ULOQ

thresholds. Vaccine response was defined as a value more than threshold (if baseline is <threshold or is missing); otherwise, it was defined as a value with a three-time increase from baseline (if baseline is ≥threshold). Group geometric mean and 95% CI are indicated.

Supplementary figure 5

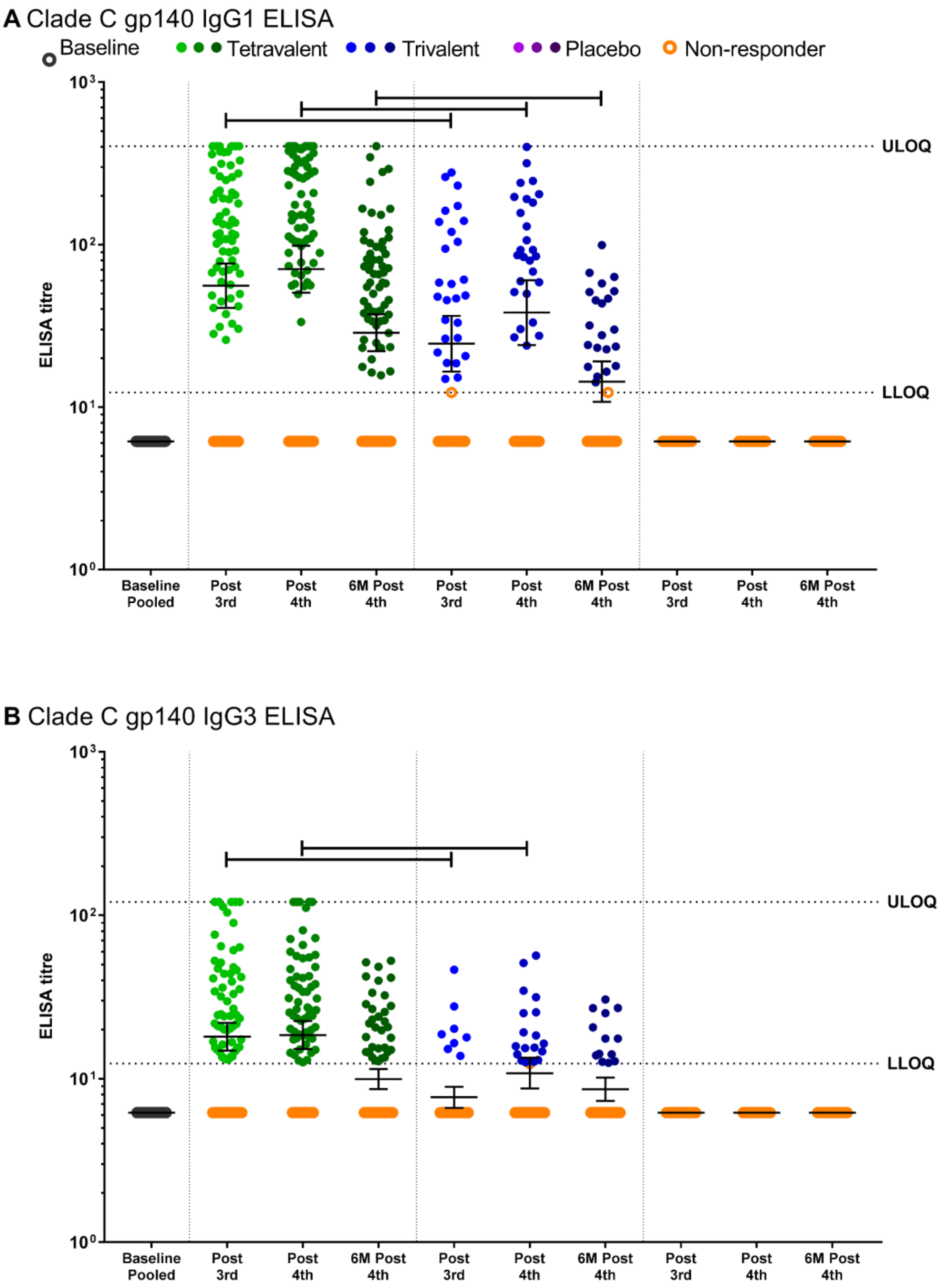

Humoral binding antibody immune responses to vaccination in humans. Response rates are shown for each vaccine group at baseline, after the third vaccination at weeks 28, after fourth vaccination at week 52 and 24 weeks after the fourth vaccination at week 72. gp140 Env Clade C ELISA IgG subclass responses for (A) IgG1 and (B) IgG3. The dotted lines are the LLOQ and ULOQ thresholds. Vaccine response was defined as value more than threshold (if baseline is < threshold or is missing); otherwise, it was defined as value with a three-time increase from baseline (if baseline is ≥ threshold). Group geometric mean and 95% CI are indicated.

Supplementary figure 6

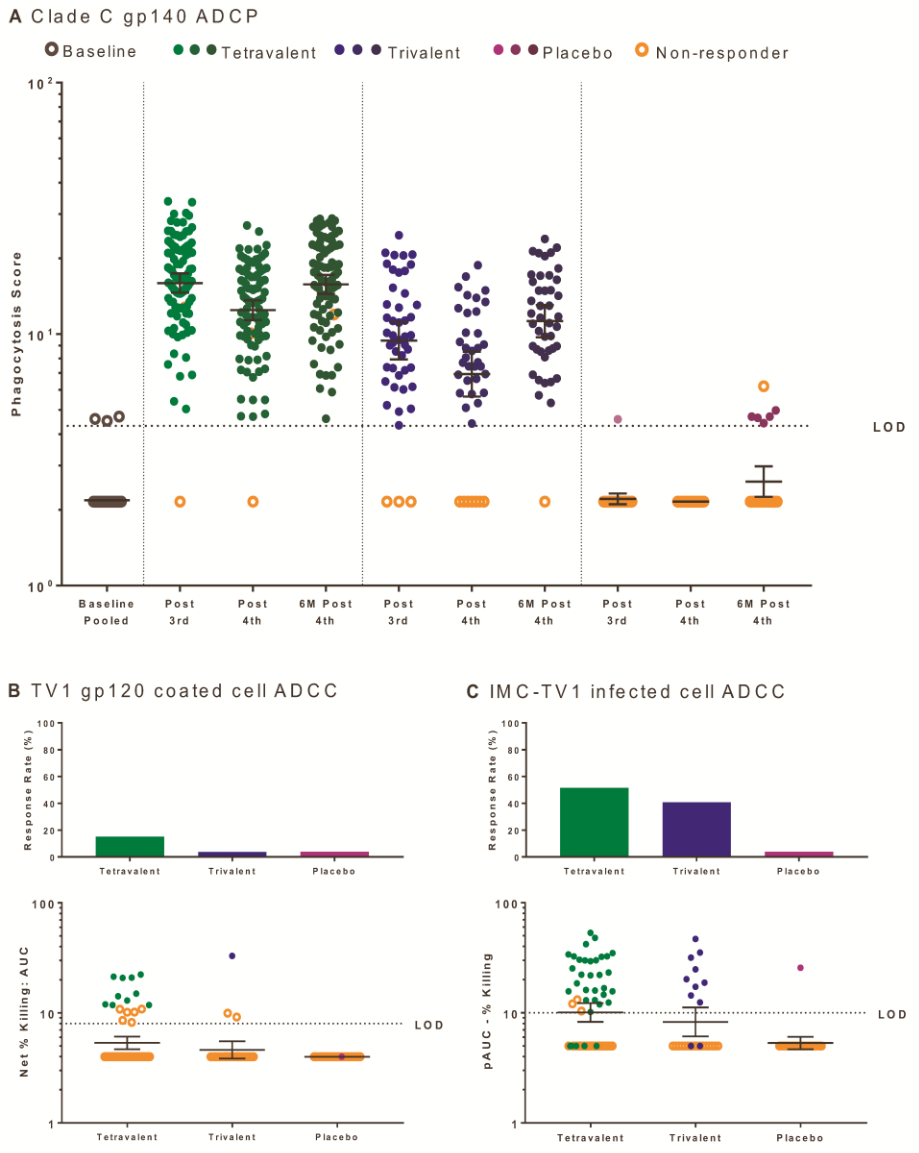

Functional humoral responses to vaccination in the per-protocol set of the three study groups: (A) Antibody dependent cellular phagocytosis of vaccine-matched Clade C gp140 Env strain 97ZA012 at the indicated time points; geometric mean ratio and 95% CI are indicated, (B) antibody dependent cellular cytotoxicity (ADCC) of

TV-1 gp120-coated target cells or (C) infectious molecular clone (IMC) TV-1 infected target cells calculated as the partial area under the curves (pAUC) determined from the baseline-subtracted ADCC percent killing response curves. ADCC responses shown are after the 3rd vaccination.

Supplementary figure 7

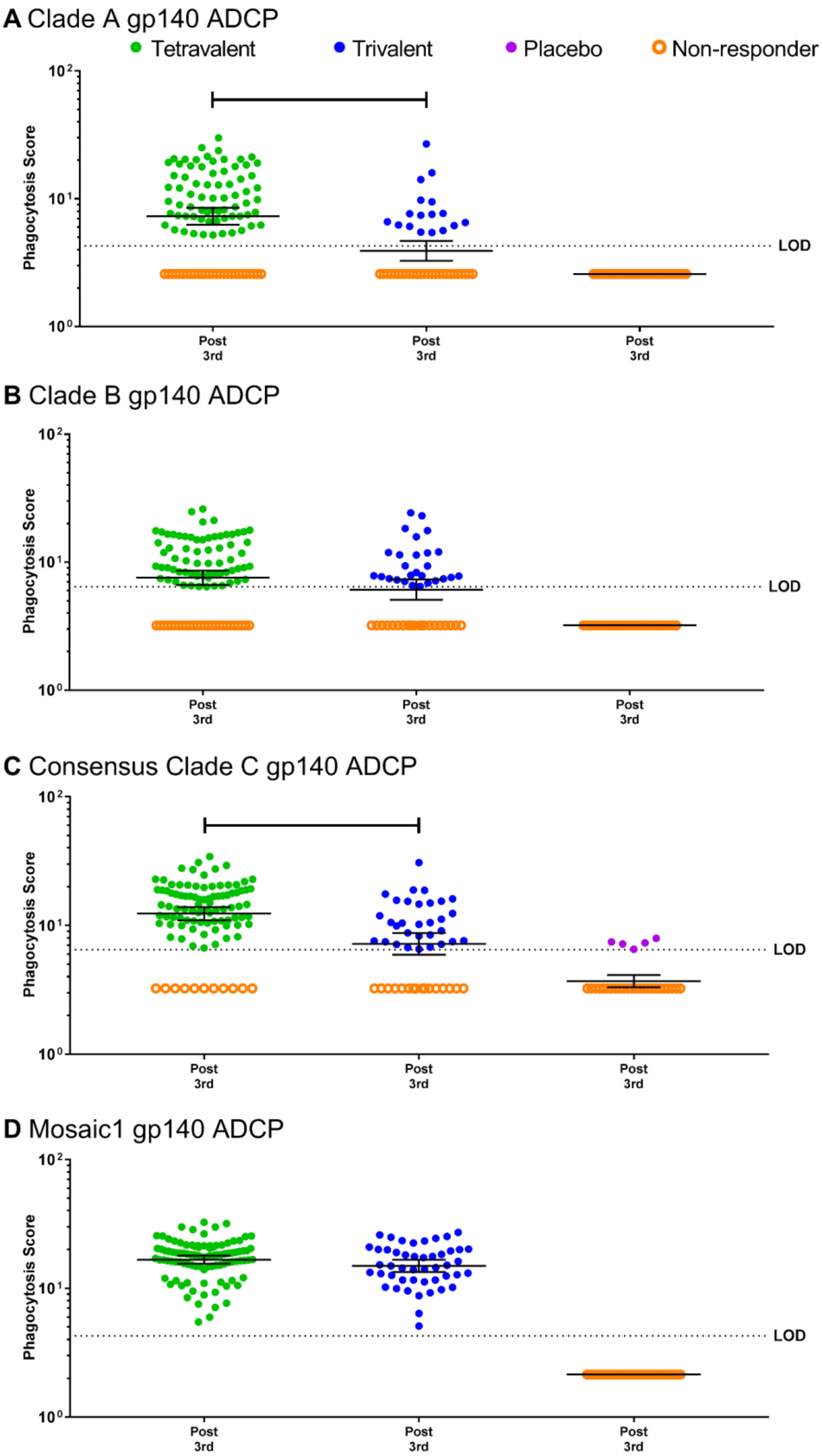

Humoral functional immune responses to vaccination. Responses are shown for each vaccine group after the third vaccination at weeks 28. gp140 Env ADCP results for (A) Clade A 92UG037.1, (B) Clade B 1990a (C) Consensus Clade C (D) Mosaic1 gp140. The dotted lines are the limits of detection thresholds. Positive responses were defined as value more than threshold. Group geometric mean and 95% CI are indicated.

Supplementary figure 8

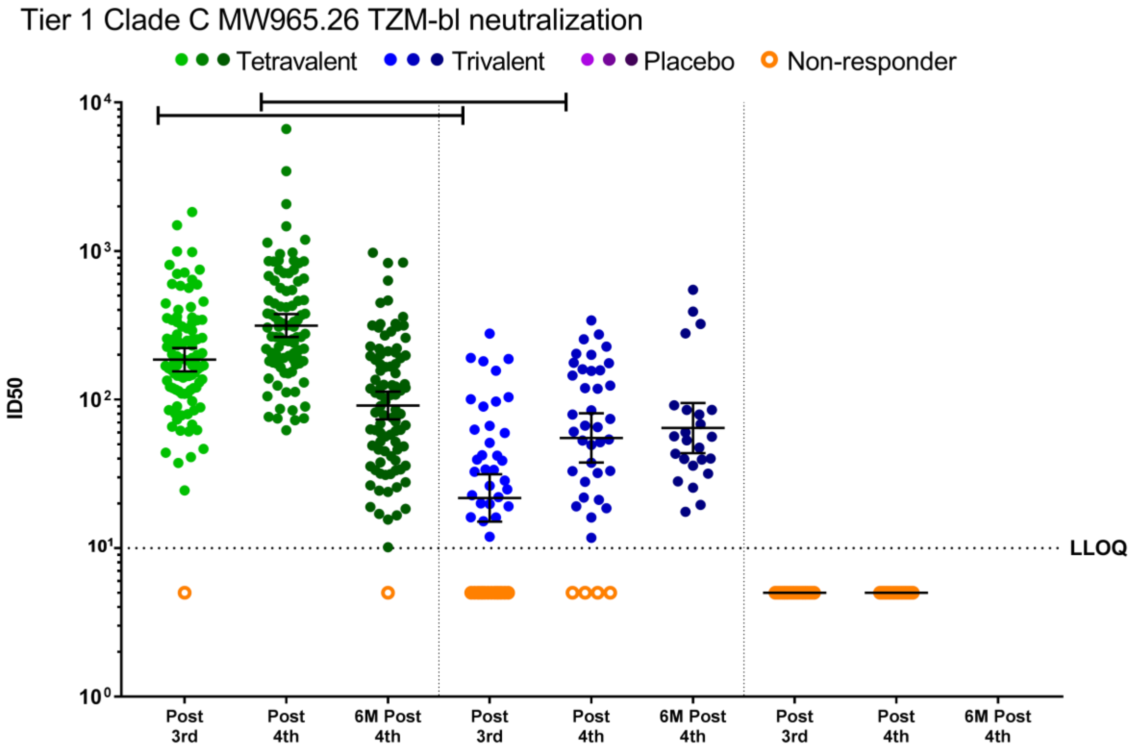

Humoral serum neutralization of MW965.26 pseudovirus responses to vaccination in the TZM-bl assay. Response rates are shown for each vaccine group after the third vaccination at weeks 28, after fourth vaccination at week 52 and 24 weeks after the fourth vaccination at week 72. The dotted lines are the LLOQ thresholds. Vaccine response was defined as a value more than threshold. Group geometric mean and 95% CI are indicated.

Supplementary figure 9

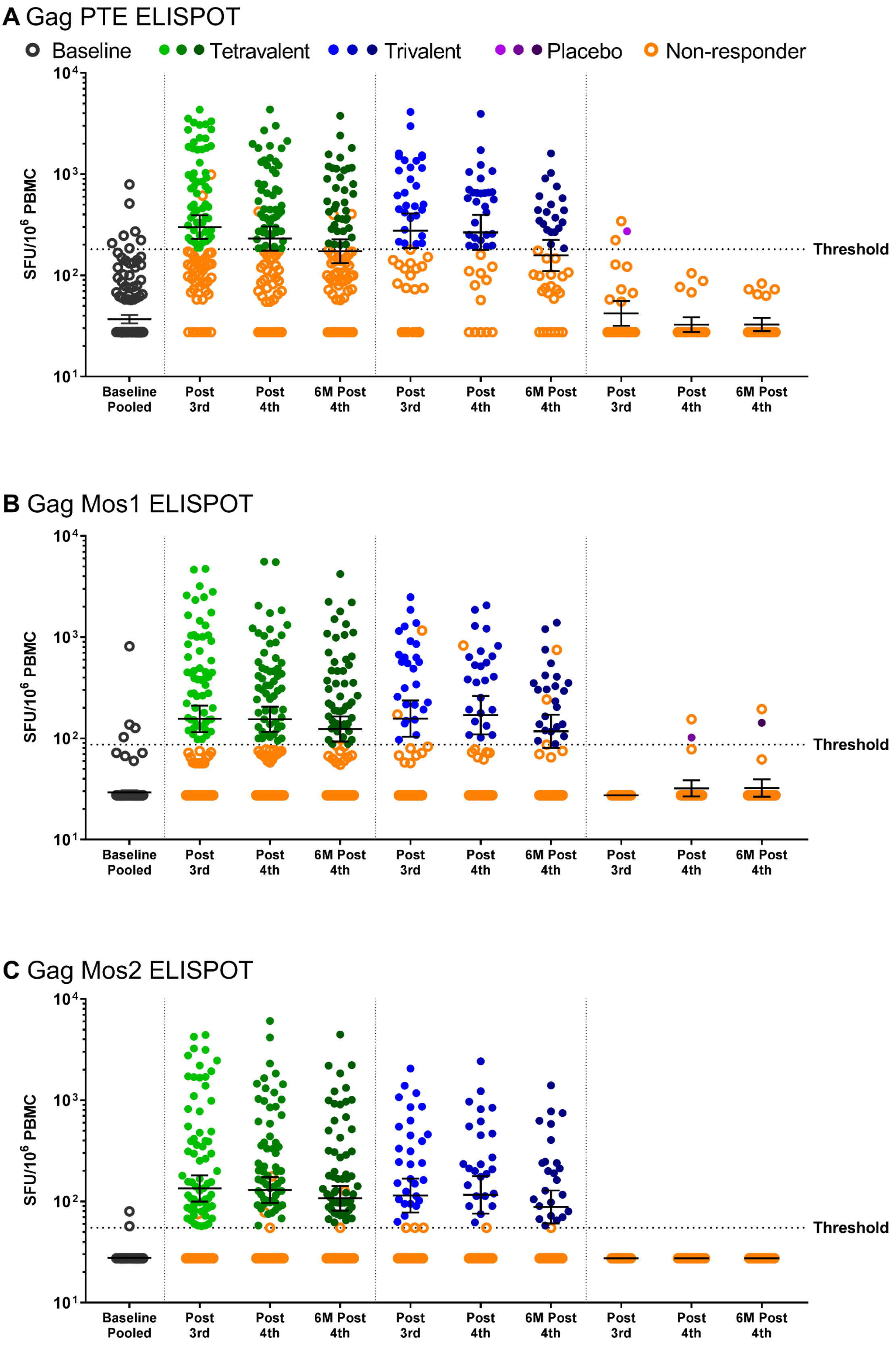

Cellular immune responses to vaccination. Response rates are shown for each vaccine group at baseline, after the third vaccination at weeks 26, after fourth vaccination at week 52 and 24 weeks after the fourth vaccination

at week 72. HIV-1 Env IFNγ specific responses for (A) Gag PTE, (B) Gag Mos1 and (C) Gag Mos2 ELISpot: The dotted line is the 95th percentile of the overall baseline values previously established.[1] Vaccine response was defined as value more than threshold (if baseline is <threshold or is missing); otherwise, it was defined as value with a three-time increase from baseline (if baseline is ≥threshold). Group median and interquartile range are indicated.

1. Barouch DH, Tomaka FL, Wegmann F, et al. Evaluation of a mosaic HIV-1 vaccine in a multicentre, randomised, double-blind, placebo-controlled, phase 1/2a clinical trial (APPROACH) and in rhesus monkeys (NHP 13-19). Lancet 2018;392:232–43.

Supplementary figure 10

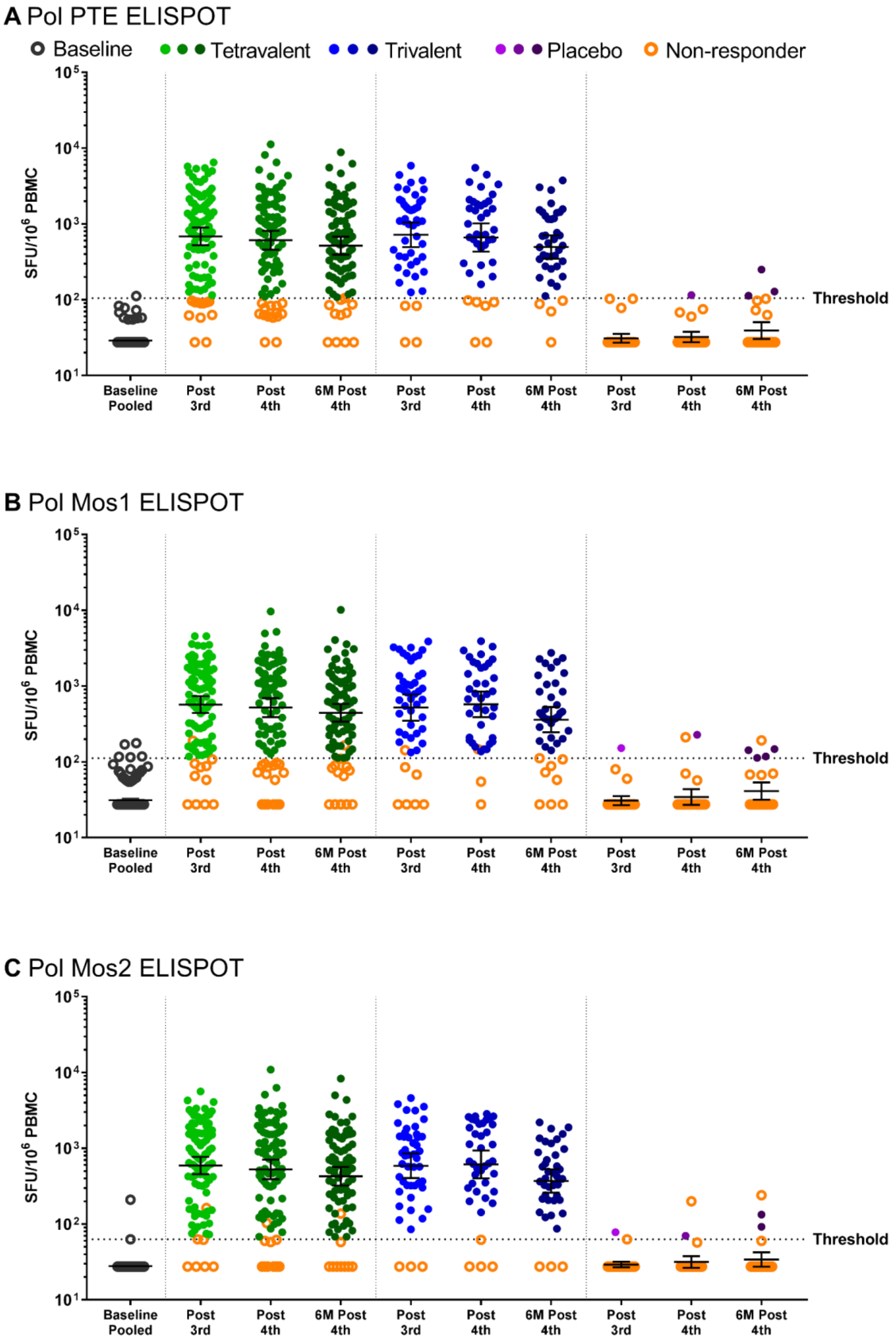

Cellular immune responses to vaccination. Response rates are shown for each vaccine group at baseline, after the third vaccination at weeks 26, after fourth vaccination at week 52 and 24 weeks after the fourth vaccination at week 72. HIV-1 Env IFNγ specific responses for (A) Pol PTE, (B) Pol Mos1 and (C) Pol Mos2 ELISpot. The

dotted line is the 95th percentile of the overall baseline values previously established.[1] Vaccine response was defined as value more than threshold (if baseline is < threshold or is missing); otherwise, it was defined as value with a three-time increase from baseline (if baseline is ≥ threshold). Group median and interquartile range are indicated.

1. Barouch DH, Tomaka FL, Wegmann F, et al. Evaluation of a mosaic HIV-1 vaccine in a multicentre, randomised, double-blind, placebo-controlled, phase 1/2a clinical trial (APPROACH) and in rhesus monkeys (NHP 13-19). Lancet 2018;392:232–43.

Supplementary figure 11

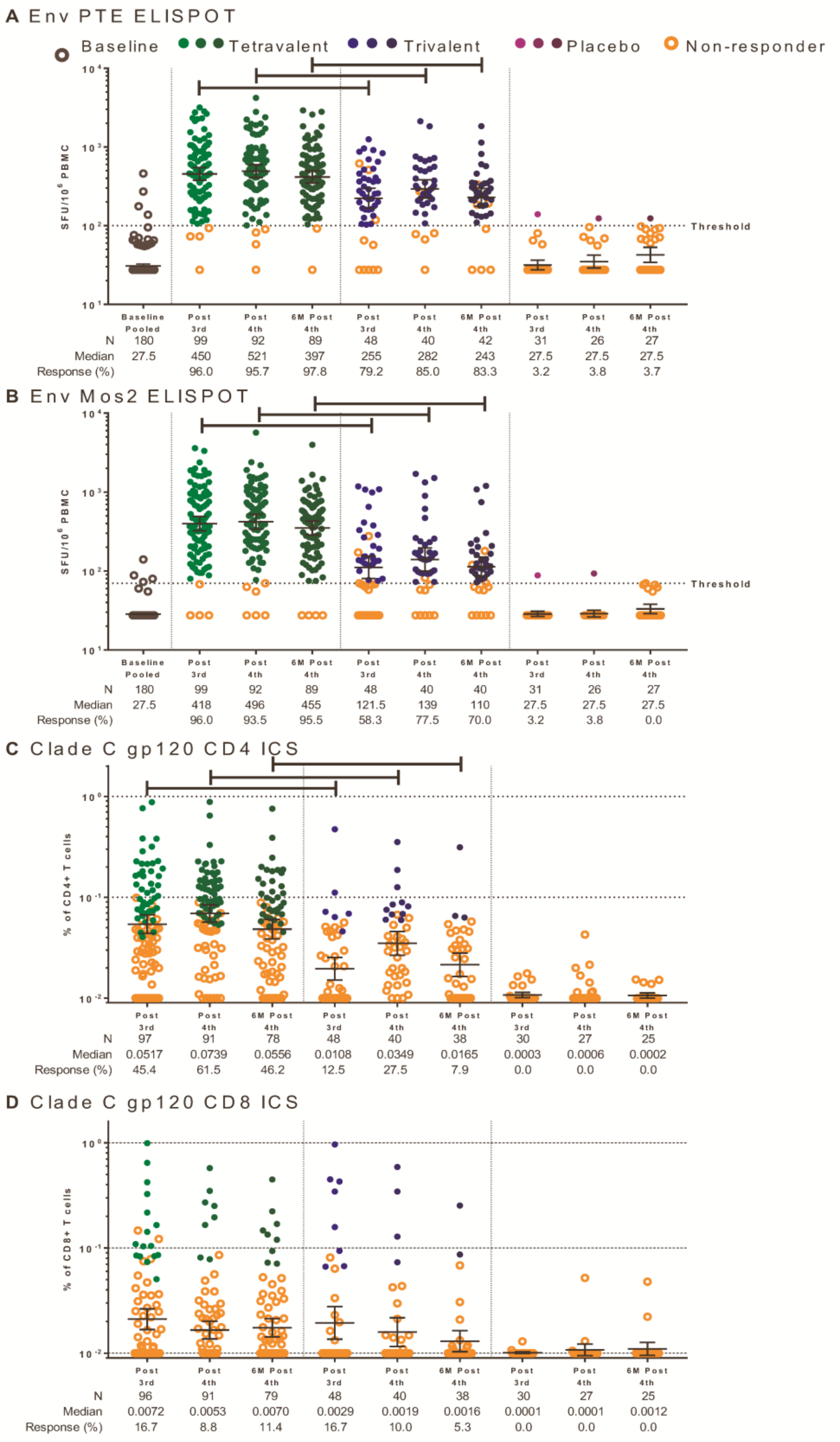

Cellular immune responses to vaccination in the three study groups at the indicated time-points in the per-protocol set: (A) Interferon-γ (IFNγ) enzyme-linked immunospot (ELISPOT) response to stimulation with potential T cell epitope (PTE), or (B) Mos2 Env peptides, or (C) intracellular cytokine staining for IFNγ and/or IL-2 production in response to Clade C gp120 by CD4 or (D) CD8 (panel D) T cells. ELISPOT responders are defined as a 3-fold increase from baseline for participants with baseline values greater than threshold, and for participants with baseline values less than threshold, post vaccination results greater than threshold. ICS responders are defined by Fisher's exact test comparison between stimulated and unstimulated cells with resultant p <1×10-5 (Per Protocol set). Indicated are the group medians and interquartile ranges of the per protocol set.

Supplementary figure 12

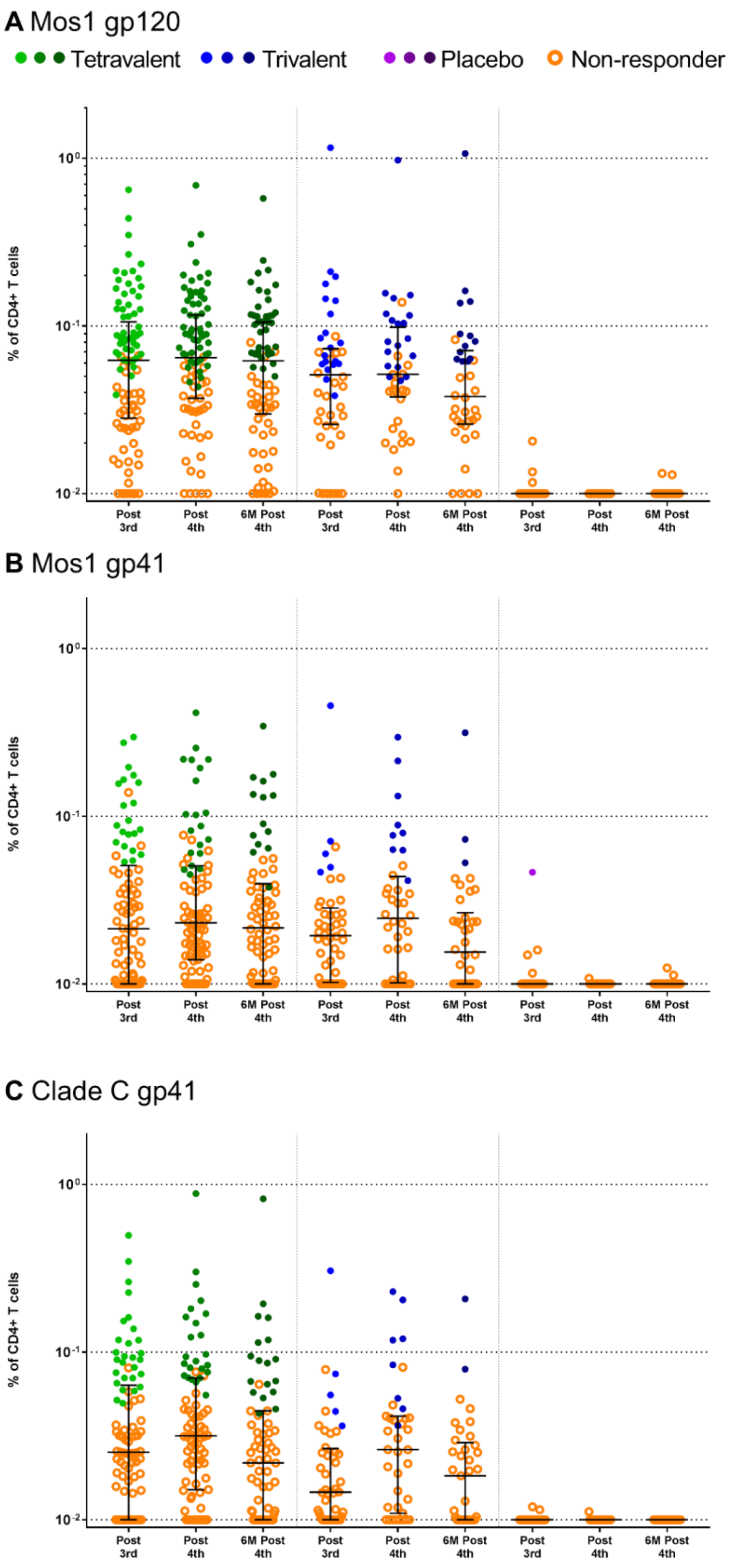

Cellular immune responses to vaccination. Response rates are shown for each vaccine group after the third vaccination at weeks 26, after fourth vaccination at week 52 and 24 weeks after the fourth vaccination at week 72. ICS for CD4 T cells producing IFNγ and/or IL-2 specific responses for (A) Mos1 gp120, (B) Mos1 gp41

and (C) Clade C gp41. Responders are defined by Fisher's exact test between stimulated and mock stimulated cells with a resultant p-value < 0.0001. Group median and interquartile range are indicated.

Supplementary figure 13

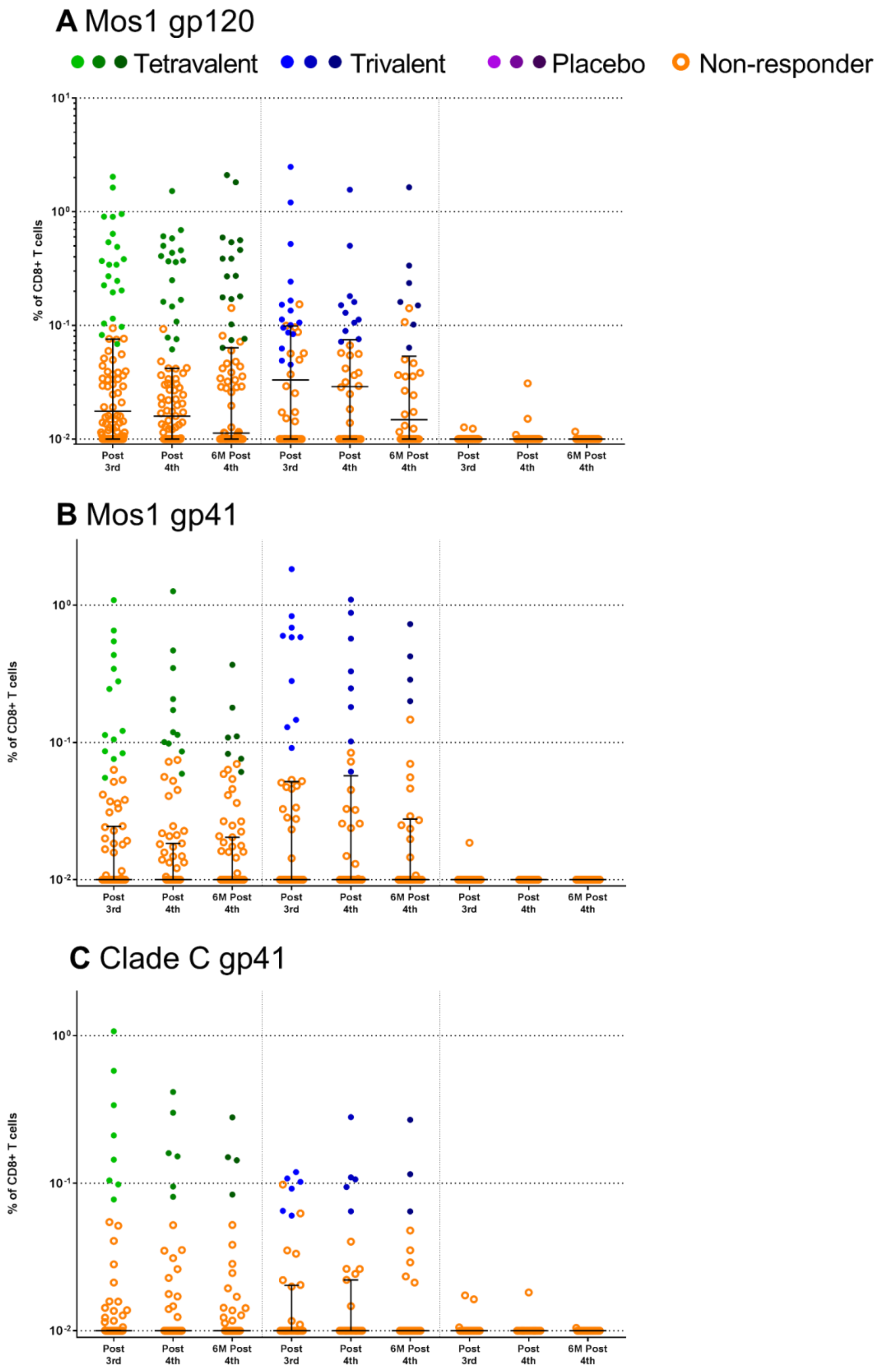

Cellular immune responses to vaccination. Response rates are shown for each vaccine group after the third vaccination at weeks 26, after fourth vaccination at week 52 and 24 weeks after the fourth vaccination at week 72. ICS for CD8 T cells producing IFNγ and/or IL-2 specific responses for (A) Mos1 gp120, (B) Mos1 gp41, (C) Clade C gp41. Responders are defined by Fisher's exact test between stimulated and mock stimulated cells with a resultant p-value < 0.0001. Group median and interquartile range are indicated.

Supplementary figure 14

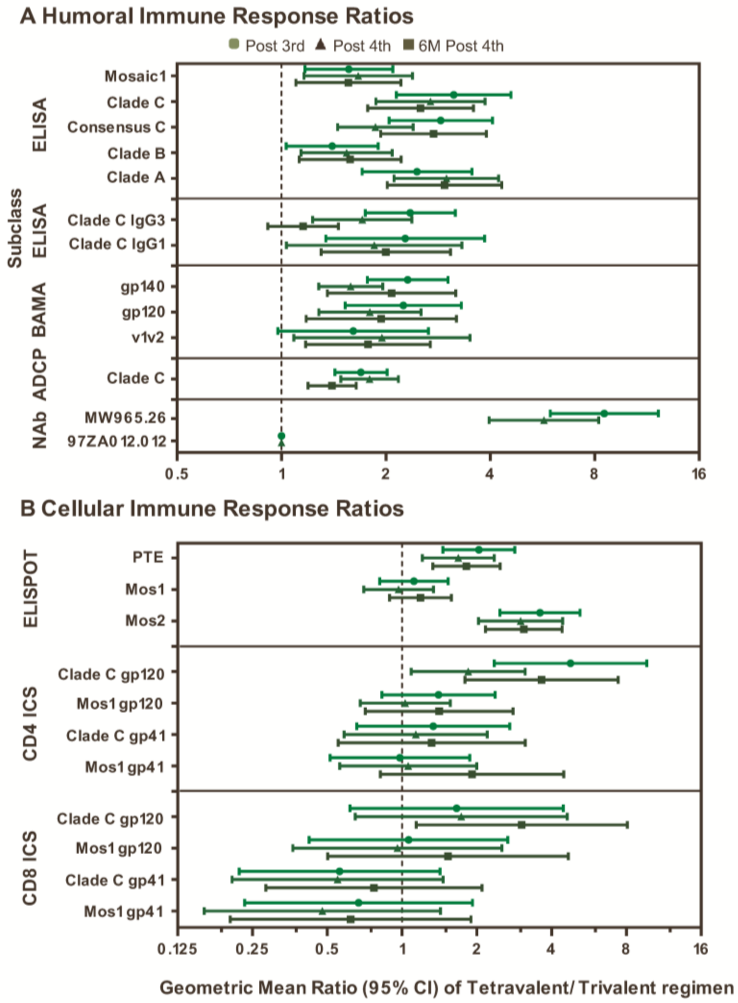

The ratio and 95% confidence intervals of (A) humoral and (B) cellular immune responses after the 3rd and 4th vaccinations and 6 months after the final vaccination. Response differences between regimens were considered statistically significant if the limit of the 95% confidence interval excluded 1.

Supplementary figure 15

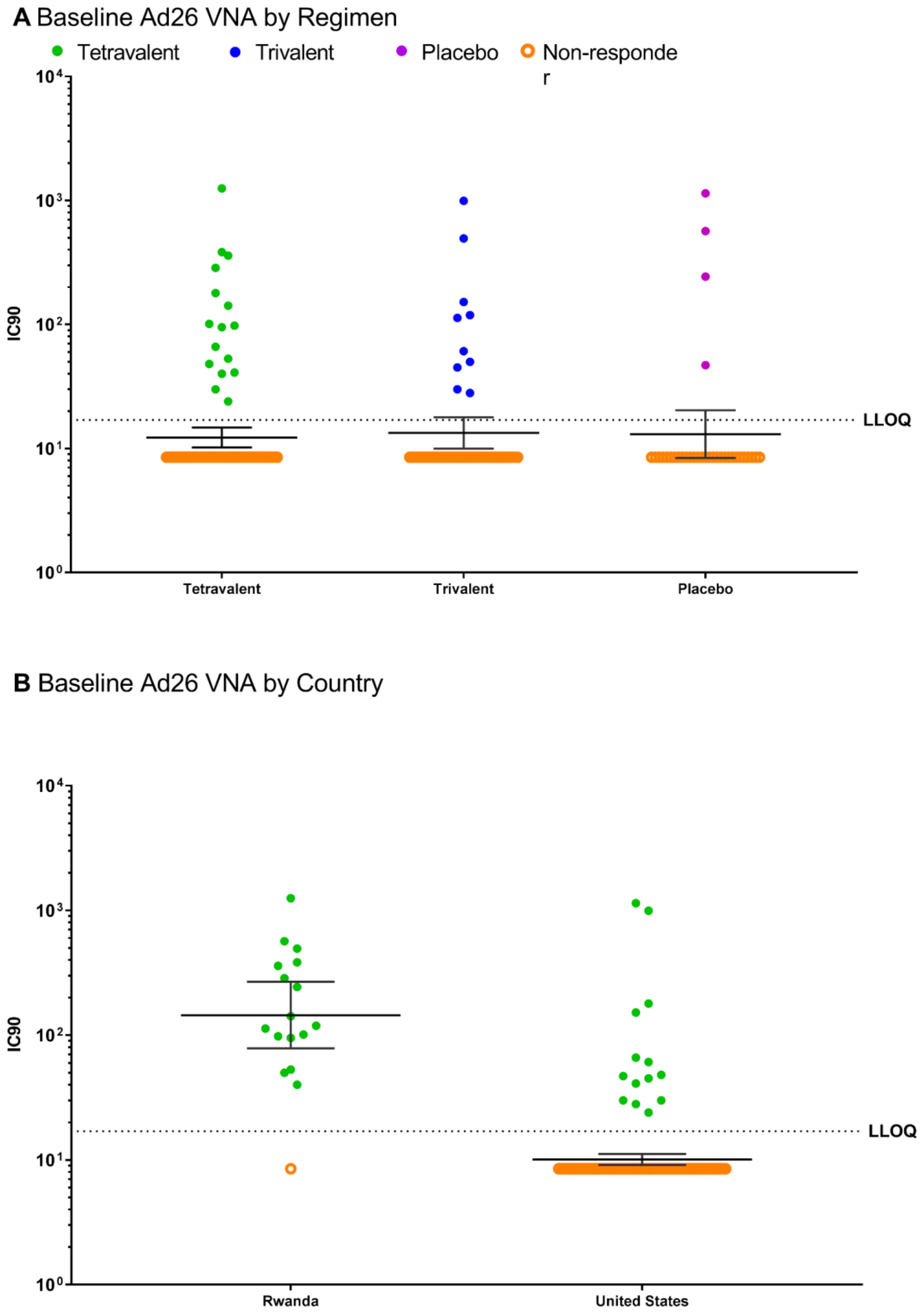

Pre-existing Ad26 neutralising antibody by (A) regimen and (B) country. Geometric mean and 95% CI are indicated.

Supplementary figure 16

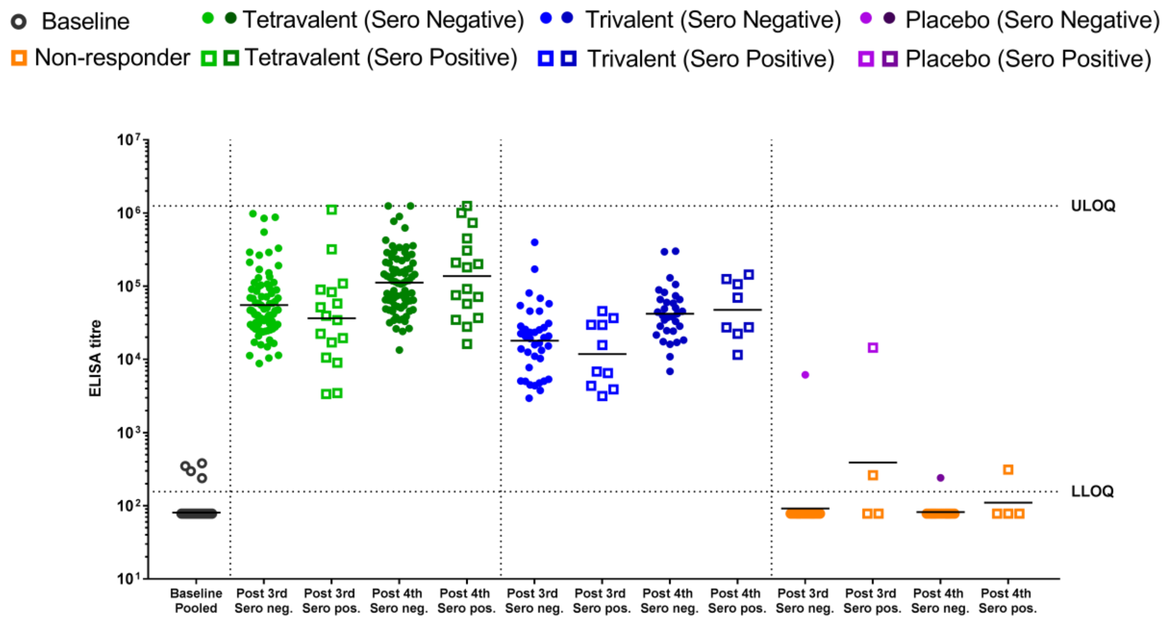

Effect of pre-existing Ad26 neutralising antibody on immune responses induced by vaccination as evaluated by total IgG Clade C gp140 ELISA. Group geometric mean is indicated.
